# Design and Synthesis of 4-Arylazo Pyrazole Carboxamides as Dual AChE/BChE Inhibitors: Kinetic and In Silico Evaluation

**DOI:** 10.3390/ph19020239

**Published:** 2026-01-29

**Authors:** Suleyman Akocak, Nebih Lolak, Hatice Esra Duran, Büşra Demir Çetinkaya, Hamada Hashem, Stefan Bräse, Cüneyt Türkeş

**Affiliations:** 1 Department of Pharmaceutical Chemistry, Faculty of Pharmacy, Adıyaman University, Adıyaman 02040, Türkiye; nlolak@adiyaman.edu.tr; 2 Department of Medical Biochemistry, Faculty of Medicine, Kafkas University, Kars 36100, Türkiye; hatice.duran@kafkas.edu.tr; 3 Department of Pharmaceutical Toxicology, Faculty of Pharmacy, Erzincan Binali Yıldırım University, Erzincan 24002, Türkiye; bdemir@erzincan.edu.tr; 4 Department of Pharmaceutical Chemistry, Faculty of Pharmacy, Sohag University, Sohag 82524, Egypt; hamada.hashem@pharm.sohag.edu.eg; 5 Institute of Biological and Chemical Systems Functional Molecular Systems (IBCS-FMS), Karlsruhe Institute of Technology (KIT), Kaiserstrasse 12, 76131 Karlsruhe, Germany; 6 Department of Biochemistry, Faculty of Pharmacy, Erzincan Binali Yıldırım University, Erzincan 24002, Türkiye

**Keywords:** pyrazole carboxamides, arylazo derivatives, acetylcholinesterase, butyrylcholinesterase, molecular docking, molecular dynamics, ADME/Tox. prediction

## Abstract

**Background/Objectives**: Pyrazole carboxamides are widely used as adaptable medicinal-chemistry scaffolds and have been explored as cholinesterase (ChE) inhibitor chemotypes. In this work, we prepared a new series of 4-arylazo-3,5-diamino-*N*-tosyl-1*H*-pyrazole-1-carboxamides **5**(**a**–**m**) and evaluated their inhibitory activity against acetylcholinesterase (AChE) and butyrylcholinesterase (BChE), supported by structure-based computational analyses. **Methods**: Thirteen derivatives **5**(**a**–**m**) were synthesized, fully characterized with analytical techniques (FT-IR, H NMR, and C NMR), and tested in vitro against AChE and BChE, with tacrine (THA) used as the reference inhibitor. Docking calculations were used to examine plausible binding modes. The top-ranked complexes (7XN1–**5e** and 4BDS–**5i**) were further examined by 100 ns explicit-solvent molecular dynamics (MD) simulations in Cresset Flare, followed by RMSD/RMSF analysis and contact-persistence profiling. Predicted ADME/Tox. properties were also assessed to identify potential developability issues. **Results**: The series showed strong ChE inhibition, and several compounds were more potent than THA. Compound **5e** (4-nitro) was the most active AChE inhibitor (*K*_I_ = 20.86 ± 1.61 nM) compared with THA (*K*_I_ = 164.40 ± 20.84 nM). For BChE, the *K*_I_ values ranged from 31.21 to 87.07 nM and exceeded the reference compound’s activity. MD trajectories supported stable binding in both systems (10–100 ns mean backbone RMSD: 2.21 ± 0.17 Å for 7XN1–**5e**; 1.89 ± 0.11 Å for 4BDS–**5i**). Most fluctuations were confined to flexible regions, while key contacts remained in place, consistent with the docking models. ADME/Tox. predictions suggested moderate lipophilicity but generally low aqueous solubility; all compounds were predicted as non-BBB permeant, and selected liabilities were flagged (e.g., carcinogenicity for **5e**/**5g**/**5h**/**5i**; nephrotoxicity for **5f**/**5g**). **Conclusions**: The 4-arylazo-3,5-diamino-*N*-tosyl-1*H*-pyrazole-1-carboxamide scaffold delivers low-nanomolar ChE inhibition, with docking and MD supporting stable binding modes. Future optimization should prioritize solubility improvement and mitigation of predicted toxicities and metabolic liabilities, especially given the predicted lack of BBB permeability for CNS-directed applications.

## 1. Introduction

Alzheimer’s disease (AD) is a leading cause of dementia and remains difficult to manage in routine clinical practice. Current estimates indicate that roughly 55 million people worldwide are affected, and projections suggest this will increase to 78 million by 2030 [1]. The disease primarily affects older adults and is characterized by a gradual decline in brain function [2]. Clinically, AD is associated with progressive memory loss, impaired learning, reduced thinking ability, and deterioration of verbal and visuospatial cognition [3,4]. As symptoms progress, patients increasingly struggle with daily activities, which ultimately compromise autonomy and quality of life. These realities continue to drive the search for more effective therapeutic options and strategies [5,6].

A consistent neurochemical hallmark of AD is the failure of cholinergic signaling. Acetylcholine (ACh) is essential for learning and memory processes, and diminished ACh availability is closely associated with cognitive decline in AD [7,8]. In parallel, cholinergic circuits are substantially damaged, further contributing to the progressive loss of cognitive function [9]. AD, however, cannot be reduced to a single pathological event; it reflects multiple interacting processes. Among the most widely emphasized are impaired cholinergic neurotransmission and β-amyloid plaque accumulation, which disrupt neuronal architecture and function [10].

**Figure 1:**
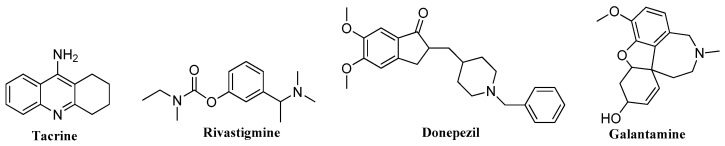
Representative FDA-approved cholinesterase inhibitors used for symptomatic management of Alzheimer’s disease.

Because of ACh depletion’s central role, cholinesterase inhibitors (ChEIs) remain a clinically established symptomatic approach. These agents inhibit acetylcholinesterase (AChE) and butyrylcholinesterase (BChE), the enzymes that hydrolyze ACh in the synaptic cleft [11,12]. By limiting ACh breakdown, ChEIs can transiently increase synaptic ACh levels and support neurotransmission, translating into modest cognitive benefits, especially in earlier disease stages [13,14]. Clinically used representatives include tacrine (THA), donepezil, rivastigmine, and galantamine (Figure 1) [15]. Even so, symptom relief is not equivalent to disease modification, and tolerability can limit long-term utility. Tacrine, for instance, was introduced early but was later withdrawn due to hepatotoxicity, illustrating the challenge of balancing efficacy and safety for this target class [16]. These limitations sustain interest in new inhibitors with improved profiles, as well as adjunct approaches that address additional AD-relevant pathways such as oxidative stress and inflammation [17,18].

**Figure 2:**
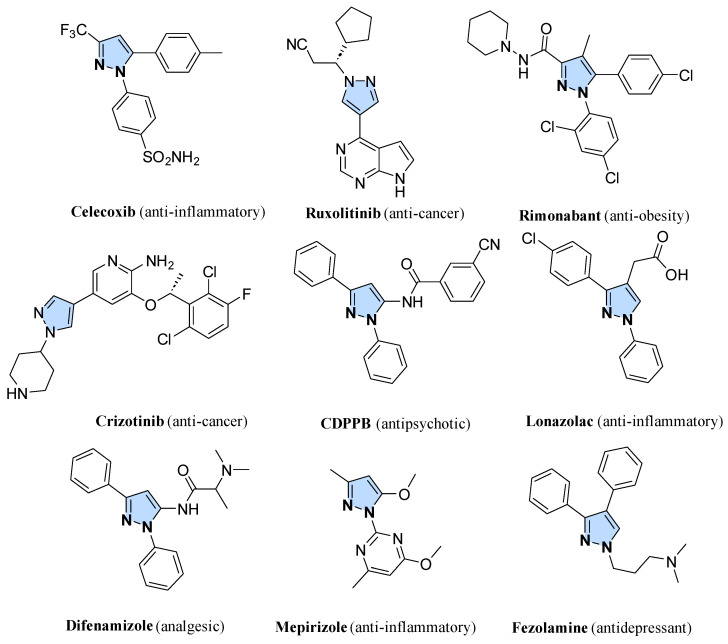
Selected clinically used small-molecule drugs featuring a pyrazole ring scaffold.

Pyrazoles are a widely used heterocyclic framework in medicinal chemistry, in part because their two adjacent ring nitrogens provide a compact platform for tuning polarity, hydrogen-bonding capacity, and lipophilicity during lead optimization [19,20,21]. The prominence of this scaffold is reflected by its presence in clinically used agents such as celecoxib, ruxolitinib, rimonabant, crizotinib, CDPPB, lonazolac, difenamizole, mepirizole, and fezolamine (Figure 2) [22]. Together, these examples illustrate the breadth of pharmacological space accessible through pyrazole-containing structures [23,24,25,26,27,28,29].

Within the broader pyrazole family, aminopyrazoles have drawn particular attention because they are adaptable across multiple application areas, including pharmaceutical research, agriculture, and dye chemistry [30]. Amino-substituted pyrazoles have been reported to display a wide range of biological activities, including antihyperglycemic, anti-inflammatory, and antibacterial effects [31,32,33,34], as well as antifungal, anticancer, antiviral, antitubercular, and antidiabetic properties [35,36,37,38]. This diversity is supported by synthetic flexibility, enabling systematic tuning of structural features relevant to bioactivity and overall performance. As an example, ATP antagonists targeting the CDK2–Cyclin E axis have been explored as oncology leads with moderate potency and encouraging selectivity [39]. In addition, 4-arylazo-3,5-diamino-1*H*-pyrazole derivatives have produced promising outcomes in various biomedical assays [40,41,42].

Despite extensive work on pyrazole-based chemotypes, comparatively fewer studies have focused on their potential as cholinesterase inhibitors. To address this gap, the present study describes the design, synthesis, and evaluation of a new series of 4-arylazo-3,5-diamino-*N*-tosyl-1*H*-pyrazole-1-carboxamide derivatives **5**(**a**–**m**). The compounds were characterized by FT-IR, H NMR, C NMR, and melting-point determination, and their inhibitory properties against ChEs were examined using both in vitro and in silico approaches to correlate potency with binding-related features. Overall, the study supports arylazopyrazole derivatives as a promising starting point for further exploration in the context of neurodegenerative disorders such as AD. In vitro cholinesterase inhibition was assessed using AChE from Electrophorus electricus and BChE from equine serum as commonly employed screening enzymes; therefore, species-related differences should be considered when extrapolating absolute potency to human cholinesterases.

## 2. Results and Discussion

### 2.1. Chemistry

**Scheme 1:**
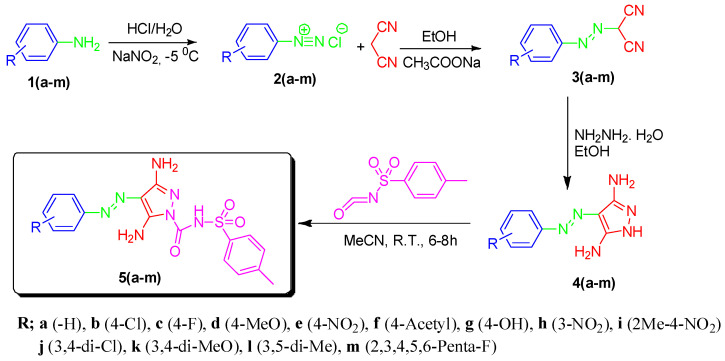
General synthetic route for the preparation of novel arylazopyrazole derivatives **5**(**a**–**m**).

The arylazopyrazole series was prepared via a short, reproducible sequence summarized in Scheme 1. The synthesis followed a protocol previously developed in our laboratory [43,44] and began with the diazotization of substituted arylamines **1**(**a**–**m**). The resulting diazonium salts were coupled with malononitrile to yield the corresponding hydrazone intermediates **3**(**a**–**m**), which were subsequently cyclized with hydrazine monohydrate to yield the pyrazole cores **4**(**a**–**m**). This ring-closure step proceeded in good yields and is consistent with related transformations reported in the literature [45,46,47,48,49]. In the final derivatization, reaction of **4**(**a**–**m**) with 4-methylbenzenesulfonyl isocyanate afforded the target 4-arylazo-3,5-diamino-*N*-tosyl-1*H*-pyrazole-1-carboxamides **5**(**a**–**m**). The formation of strongly colored products was taken as an experimental indicator of successful azo incorporation. The crude solids were isolated by filtration and washed thoroughly with diethyl ether to remove residual reagents and byproducts, providing clean materials suitable for downstream biological testing.

Structural confirmation of the final compounds was achieved by FT-IR, H NMR, and C NMR spectroscopy, supported by melting point measurements. The H NMR spectra of all assessed substances were obtained employing tetramethylsilane (TMS) as the internal standard in DMSO-d_6_ as the solvent. The consequent findings are further discussed in the experimental phase of the study. The existence of the urea group in all produced compounds **5**(**a**–**m**) has been confirmed by a distinctive singlet signal (9.41–9.51 ppm) corresponding to the amidic proton. In all compounds, the protons in the aromatic rings (ArC–H) exhibited resonances as singlets, doublets, and multiplets in the δ 8.01–8.28, δ 7.66–7.90, δ 7.50–7.58, and δ 7.14–7.48 ppm ranges, respectively. In compounds **5**(**a**–**m**), all –CH_3_ peaks on the aromatic ring were detected in the δ 2.27–2.28 ppm range. The particular –NH_2_ peaks are first found in the δ 7.80–8.20 ppm range, followed by the second in the δ 6.09–6.28 ppm region. In compounds **5d** and **5k**, the (–OCH_3_) protons are detected as a singlet in the δ 3.80–3.83 ppm range. Conversely, for compounds **5f**, **5i**, and **5l**, the –CH_3_ protons were detected at δ 2.59, 2.57, and 2.32 ppm, respectively. The acquired data aligned with literature values, and the integral ratios corresponded with the theoretically predicted number of protons. Therefore, it was determined that the compounds with the suggested structures were produced effectively.

The C NMR spectra of all analyzed substances were collected utilizing tetramethylsilane (TMS) as the internal standard in DMSO-d_6_ as the solvent. The C NMR spectra of the produced compounds revealed the characteristic signal of the carbonyl carbon in the ureido group within the 150.33–159.43 ppm range. The chemical shifts of carbon atoms in the aromatic rings of compounds **5**(**a**–**m**) were recorded between 150.58 and 102.32 ppm. The methyl (–CH_3_) group on the phenyl ring of all substances was found at 20.89–20.90 ppm. The methyl (-CH_3_) groups of molecules **5f**, **5i**, and **5l** were observed at 27.21, 18.15, and 21.43 ppm, respectively. On the other hand, the distinct methoxy (–OCH_3_) peaks of compounds **5d** and **5k** were detected at 55.83, 55.98, and 56.08 ppm, respectively. These findings align with literature values and corroborate the hypothesized structures.

### 2.2. Biological Evaluation

To translate the synthetic effort into a functional readout, the **5**(**a**–**m**) series was prioritized for cholinesterase profiling because the shared *N*-tosyl-pyrazole-1-carboxamide core offers a compact hydrogen-bonding framework. At the same time, the arylazo fragment provides an extended aromatic surface and enables systematic tuning through terminal-aryl substitution. This matched-series design allows for discussion of activity differences primarily in terms of substituent effects on the terminal ring, while keeping the remainder of the scaffold constant.

**Figure 3:**
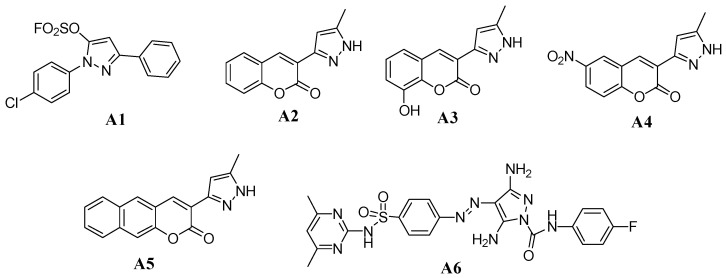
Selected pyrazole-based compounds reported in the literature have potent ChE inhibitory activity. Reported potencies and inhibition behavior (reversible vs. time-dependent) are summarized at the order-of-magnitude level based on the cited references.

Guided by prior pyrazole-derived ChEI studies (Figure 3), we selected AChE and BChE inhibition as the primary biological endpoint and complemented the enzymatic dataset with structure-based analyses. Docking was employed to propose binding orientations and key contacts for representative high-activity members, and explicit-solvent MD simulations were used to assess pose stability and interaction persistence over time for the top-ranked complexes. In addition, cytotoxicity screening and in silico ADME/Tox profiling were included to provide an early view of safety- and developability-related considerations alongside enzyme potency. A limitation is that inhibition constants were obtained using non-human enzyme preparations; therefore, confirmatory testing against human AChE/BChE will be required in follow-up work to strengthen translational interpretation.

Literature precedents indicate that pyrazole-based scaffolds can be tuned toward cholinesterase targets (Figure 3). For example, Li et al. [50] reported fluorosulfate-substituted pyrazoles (e.g., **A1**) with measurable AChE/BChE inhibition. They discussed possible time-dependent/reactive behavior, while coumarin–pyrazole hybrids have shown potencies comparable to reference inhibitors under the respective assay conditions. Coumarin–pyrazole hybrids (**A2**–**A5**) have likewise been described with inhibitory potencies comparable to reference compounds under the same study conditions [51]. In our earlier work, sulfonamide-derived pyrazole carboxamides (e.g., **A6**) were reported to be potent, reversible dual ChEIs in the low-nanomolar range [52]. Together, these reports motivated evaluation of the present **5**(**a**–**m**) series; however, confirmatory profiling against human AChE/BChE will be required in follow-up studies to strengthen translational interpretation.

#### 2.2.1. In Vitro Cholinesterase Inhibition Profile

Compounds **5**(**a**–**m**) were evaluated in vitro against AChE and BChE, with THA included as the reference inhibitor (Table 1). All derivatives inhibited both enzymes at low nanomolar concentrations. For AChE, *K*_I_ values extended from 20.86 ± 1.61 nM to 46.07 ± 2.31 nM. For BChE, *K*_I_ values ranged from 31.21 ± 2.65 nM to 87.07 ± 5.67 nM. Under the same assay conditions, THA was notably less potent (AChE *K*_I_ = 164.40 ± 20.84 nM; BChE *K*_I_ = 341.80 ± 53.35 nM). In this dataset, therefore, each member of the **5**(**a**–**m**) series outperformed the reference compound. Model fitting was consistent across the dataset, yielding high coefficients of determination (*R*^2^ ≈ 0.986–0.989), which supports the reliability of the calculated *K*_I_ values. Given the sensitivity of THA potency to enzyme source and assay conditions, THA is used here primarily as an internal reference assayed alongside all compounds **5**(**a**–**m**) under identical conditions.

#### 2.2.2. Most Active Compounds and Enzyme Preference

**Figure 4:**
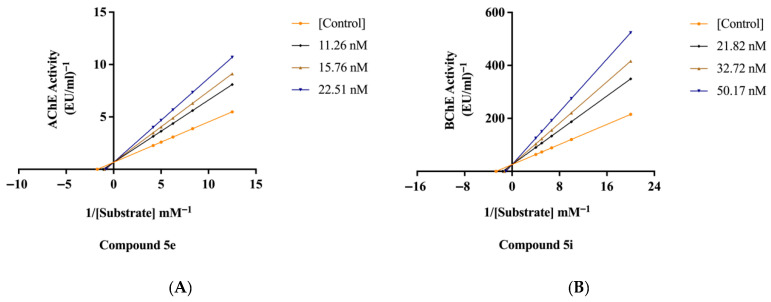
Lineweaver–Burk analysis of cholinesterase inhibition for (**A**) AChE in the presence of 3,5-diamino-4-[(4-nitrophenyl)diazenyl]-*N*-tosyl-1*H*-pyrazole-1-carboxamide (**5e**) and (**B**) BChE in the presence of 3,5-diamino-4-[(2-methyl-4-nitrophenyl)diazenyl]-*N*-tosyl-1*H*-pyrazole-1-carboxamide (**5i**). Double-reciprocal plots are shown (*y*-axis: 1/v, where v is the initial rate, ΔOD_412/min_; *x*-axis: 1/[S]). The corresponding secondary replots (slope vs. inhibitor concentration and, where appropriate intercept vs. inhibitor concentration) used to estimate *K*_I_ are provided in the accompanying panel/insets.

Within the AChE dataset, **5e** (4-nitro analogue) was the most potent inhibitor, exhibiting the lowest *K*_I_ (20.86 ± 1.61 nM). Several additional compounds followed closely, including **5k** (27.04 ± 2.10 nM) and **5m** (27.20 ± 2.20 nM), as well as **5h** (28.78 ± 2.28 nM) and **5d**/**5f** (~30 nM). This clustering indicates that multiple terminal-aryl substitution patterns can retain high AChE affinity within the same scaffold. Representative inhibition curves for **5e** are shown in Figure 4.

For BChE, the strongest inhibition was observed for **5i** (31.21 ± 2.65 nM) and **5c** (31.45 ± 2.80 nM). Notably, **5c** combined top-tier BChE potency with a comparatively weaker AChE value (42.48 ± 3.36 nM), suggesting a shift toward BChE preference for that substitution pattern. A similar, though less pronounced, bias was also evident for **5i** (AChE *K*_I_ = 38.37 ± 2.05 nM; BChE *K*_I_ = 31.21 ± 2.65 nM). Representative inhibition curves for **5i** are provided in Figure 4.

To summarize enzyme preference within this matched set, BChE/AChE *K*_I_ ratios were calculated (values < 1 indicate relatively stronger BChE inhibition). In this comparison, **5c** showed the clearest BChE-leaning profile (~0.74, i.e., ~1.35-fold stronger inhibition of BChE than AChE), followed by **5i** (~0.81, ~1.23-fold BChE-favored). In contrast, **5e** was distinctly AChE-leaning (~4.17), reflecting a strong AChE *K*_I_ alongside comparatively weaker BChE inhibition.

#### 2.2.3. Structure–Activity Observations Within the **5**(**a**–**m**) Set

All members of the series share the same *N*-tosyl-pyrazole-1-carboxamide core and differ primarily at the terminal aryl unit introduced through the arylazo (–N=N–Ar) linkage. This design enables a focused view of how terminal-ring substitution influences ChE inhibition with minimal scaffold-level confounding. The set includes an unsubstituted phenyl analogue (**5a**), halogenated aryls (**5b**, **5c**, **5j**, **5m**), electron-donating substituents (**5d**, **5g**, **5k**, **5l**), and electron-withdrawing motifs (**5e**, **5f**, **5h**, **5i**). Three trends are apparent from the present data:

(i) Nitro-containing analogues are prominent among the strongest AChE inhibitors (e.g., **5e** and **5h**), indicating that strongly electron-withdrawing terminal substitution is compatible with high AChE affinity in this scaffold family.

(ii) The leading BChE inhibitors (**5c** and **5i**) show that BChE potency can be optimized within the same chemical platform and that specific substitution patterns can shift enzyme preference away from AChE.

(iii) Introduction of a phenolic group (**5g**) coincided with the weakest AChE potency in the set (AChE *K*_I_ = 46.07 ± 2.31 nM) while maintaining BChE inhibition in the same low-nanomolar regime (BChE *K*_I_ = 44.56 ± 3.94 nM).

**Figure 5:**
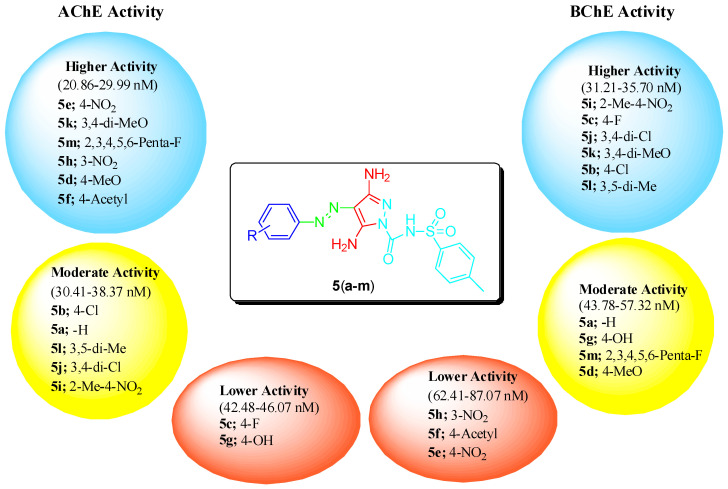
Structure–activity relationship (SAR) summary for the synthesized 4-arylazo-3,5-diamino-*N*-tosyl-1*H*-pyrazole-1-carboxamides **5**(**a**–**m**), derived from the matched series and their in vitro inhibitory activities against AChE and BChE.

These substituent-linked differences support a tunable inhibition profile across the series and are summarized in the schematic SAR (Figure 5).

Overall, the **5**(**a**–**m**) series exhibited consistently strong inhibition of both ChEs, with all analogues outperforming THA under the present assay conditions (Table 1). The dataset also reveals substituent-driven tuning of enzyme preference: **5e** is the strongest AChE inhibitor, whereas **5c** and **5i** exhibit the most potent BChE inhibition and show a relative BChE bias. Together, these findings position the arylazopyrazole carboxamide framework as a solid starting point for further optimization toward potent, and potentially enzyme-selective, ChEIs.

#### 2.2.4. Cytotoxicity Effects on Normal and Cancer Cell Lines

Compounds **5**(**a**–**m**) were evaluated for cytotoxicity using the MTT assay, and IC_50_ values were determined in three human cell lines: Beas-2B (normal bronchial epithelial), A549 (lung carcinoma), and MCF-7 (breast adenocarcinoma). 5-Fluorouracil (5-FU), a clinically used antimetabolite, was included as the reference compound [53,54]. To estimate preferential activity toward malignant cells, a selectivity index (*S*_I_) was calculated as *S*_I_ = IC_50_(Beas-2B)/IC_50_(cancer), where *S*_I_ > 1 indicates greater selectivity toward the cancer line [55]. The IC_50_ values and derived *S*_I_ metrics are summarized in Table 2.

Across the panel (Table 2), changing the terminal aryl group within this azo-linked series produced clear shifts in both cytotoxic potency and apparent selectivity. Because the core scaffold is conserved, the differences are most reasonably attributed to substituent-driven effects on physicochemical features (e.g., polarity and hydrogen-bonding capacity), which can influence cellular exposure and non-selective toxicity.

Among the evaluated compounds, **5c** (p-fluoro) showed the most favorable overall balance. It displayed moderate activity in both cancer lines (A549 IC_50_ = 139.53 µM; MCF-7 IC_50_ = 113.76 µM) while remaining substantially less cytotoxic in the normal line (Beas-2B IC_50_ = 502.47 µM). As a result, **5c** produced the highest selectivity indices in the dataset (*S*_I_ = 3.60 for Beas-2B/A549 and 4.42 for Beas-2B/MCF-7). In contrast, the unsubstituted analogue **5a** showed weaker performance overall and no measurable MCF-7 value (ND; not determined), consistent with the idea that terminal-ring substitution can be important for tuning phenotype-level outcomes in this series.

Increased potency did not necessarily translate into a useful window. The p-methoxy derivative **5d** was active in MCF-7 (IC_50_ = 28.18 µM) but was even more cytotoxic in Beas-2B (IC_50_ = 10.36 µM), giving poor selectivity (Beas-2B/MCF-7 *S*_I_ = 0.37). A related pattern was observed for the p-hydroxy analogue **5g**, which was the most active compound in A549 (IC_50_ = 26.98 µM) yet showed limited selectivity because Beas-2B cytotoxicity was comparable (Beas-2B IC_50_ = 28.28 µM; Beas-2B/A549 *S*_I_ = 1.05). Together, these observations suggest that strongly hydrogen-bonding substituents (e.g., OMe, OH) may improve potency in a cell-line-dependent manner but can also narrow selectivity through concomitant toxicity in the normal line.

Halogenation effects were more variable. The p-chloro compound **5b** exhibited modest potency and only moderate selectivity toward MCF-7 (Beas-2B/MCF-7 *S*_I_ = 1.55). For **5j** (dichloro) and **5m** (pentafluorophenyl), interpretation was limited by ND entries in one or more cancer lines, which prevents firm ranking of these substituent classes across the full panel.

Electron-withdrawing substituents showed divergent effects across cancer models. The nitro derivative **5e** displayed a pronounced reduction in activity in MCF-7 (IC_50_ = 1245.98 µM) while retaining modest activity in A549 (IC_50_ = 236.01 µM). This translates into an A549/MCF-7 ratio of 0.19, but also yields poor selectivity versus the normal line (Beas-2B/A549 *S*_I_ = 0.40; Beas-2B/MCF-7 *S*_I_ = 0.08). For the nitro-containing variants **5h** and **5i**, MCF-7 values were ND, restricting broader comparison; however, **5i** showed a favorable A549 selectivity index (*S*_I_ = 3.28) driven by A549 IC_50_ = 82.96 µM relative to Beas-2B IC_50_ = 272.25 µM, suggesting that some nitro-bearing patterns may retain cancer preference in the A549 model.

Other substituent classes produced mixed profiles. The p-acetyl analogue **5f** was weakly active (A549 IC_50_ ≈ 492.63 µM; MCF-7 IC_50_ = 258.05 µM) but showed moderate selectivity toward MCF-7 (Beas-2B/MCF-7 *S*_I_ = 1.93). The dimethoxy compound **5k** and the dimethyl compound **5l** showed limited selectivity toward MCF-7 (Beas-2B/MCF-7 *S*_I_ = 0.23 and 0.72, respectively), indicating that additional substitution or increased bulk does not inherently improve cancer preference in this dataset.

Compared with 5-FU, which displayed broadly similar cytotoxicity across the tested lines and no selectivity toward A549 (*S*_I_ = 0.86), the most notable outcome is the performance of **5c**, which combines measurable activity with the highest selectivity indices across both cancer models. On this basis, **5c** is the clearest candidate for prioritization within the present panel. At the same time, completing the ND measurements would be important to strengthen substitution-level conclusions for several analogues.

Overall, terminal aryl substitution strongly modulated cytotoxicity and selectivity. The p-fluoro analogue **5c** provided the most favorable balance (highest *S*_I_ values for both A549 and MCF-7). In contrast, hydrogen-bonding substituents (e.g., **5d** and **5g**) tended to increase potency in a cell-line-dependent manner but generally reduced selectivity due to parallel Beas-2B toxicity. Several multi-substituted and nitro-containing analogues remain only partially interpretable because of ND IC_50_ values.

### 2.3. Molecular Docking Study

Molecular docking was used to examine how the newly synthesized 4-arylazo-3,5-diamino-*N*-tosyl-1*H*-pyrazole-1-carboxamides **5**(**a**–**m**) can be accommodated within the binding sites of AChE and BChE, with attention to pose plausibility and selectivity-related features. Before analyzing the test ligands, the docking protocol was validated by re-docking the co-crystallized reference inhibitor THA into the corresponding crystal structures (AChE: PDB ID 7XN1 [56]; BChE: PDB ID 4BDS [57]). In both cases, the re-docked poses closely reproduced the experimental binding geometry, with RMSD values below 0.3 Å, supporting the reliability of the workflow for subsequent interaction analysis.

Within the series, **5e** (experimentally identified as a competitive AChE inhibitor) showed the most favorable docking behavior in AChE, yielding a docking score of −7.52 kcal/mol. The predicted pose, positioned **5e** along the aromatic gorge, and was stabilized by complementary non-covalent contacts. A prominent feature was π–π stacking with Trp86, consistent with the role of aromatic anchoring interactions in this enzyme. In addition, the pyrazole ring formed a hydrogen bond with Tyr124 at 1.97 Å, providing an extra stabilizing contribution. Together, these interactions support a binding mode compatible with competitive inhibition, and suggest clear opportunities for substitution-driven tuning through modifications to the nitrophenyl and/or pyrazole fragments.

**Figure 6:**
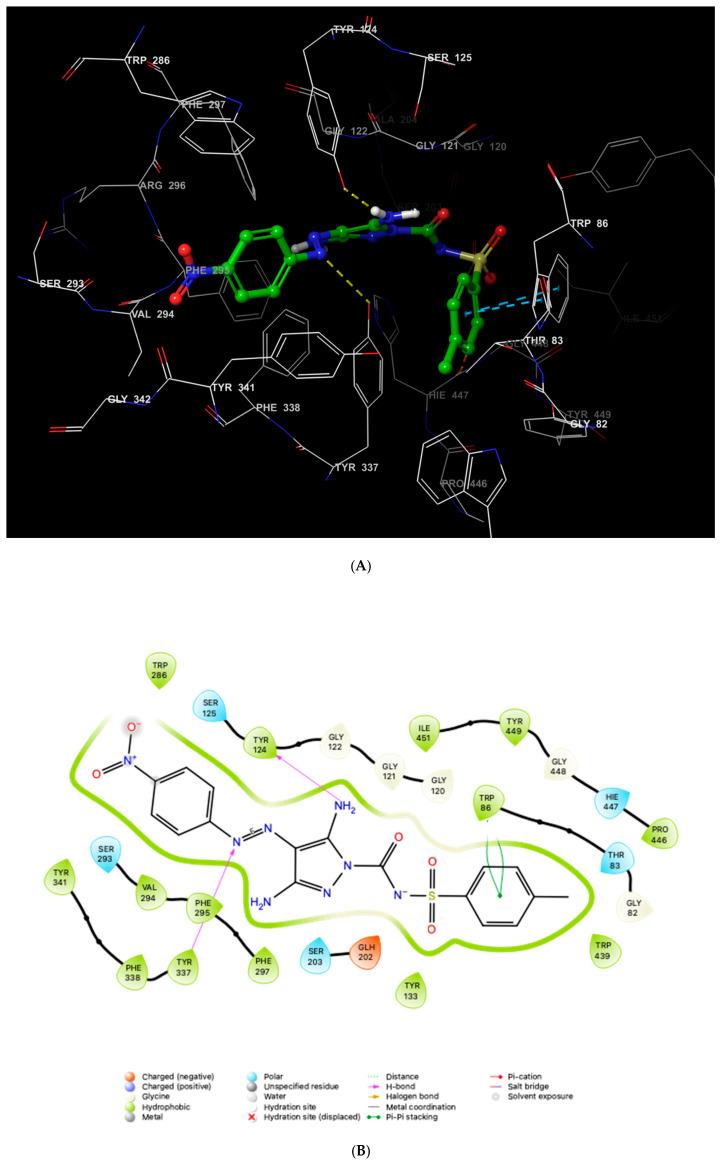
Docking pose of **5e** in AChE (PDB ID: 7XN1). (**A**) Predicted 3D binding orientation within the active-site gorge (H-bonds: yellow dashed lines; π–π interactions: blue dashed lines). (**B**) 2D interaction diagram showing key contacts. Key residues were labelled for orientation, and the catalytic serine (Ser203) is emphasized in both panels.

**Figure 7:**
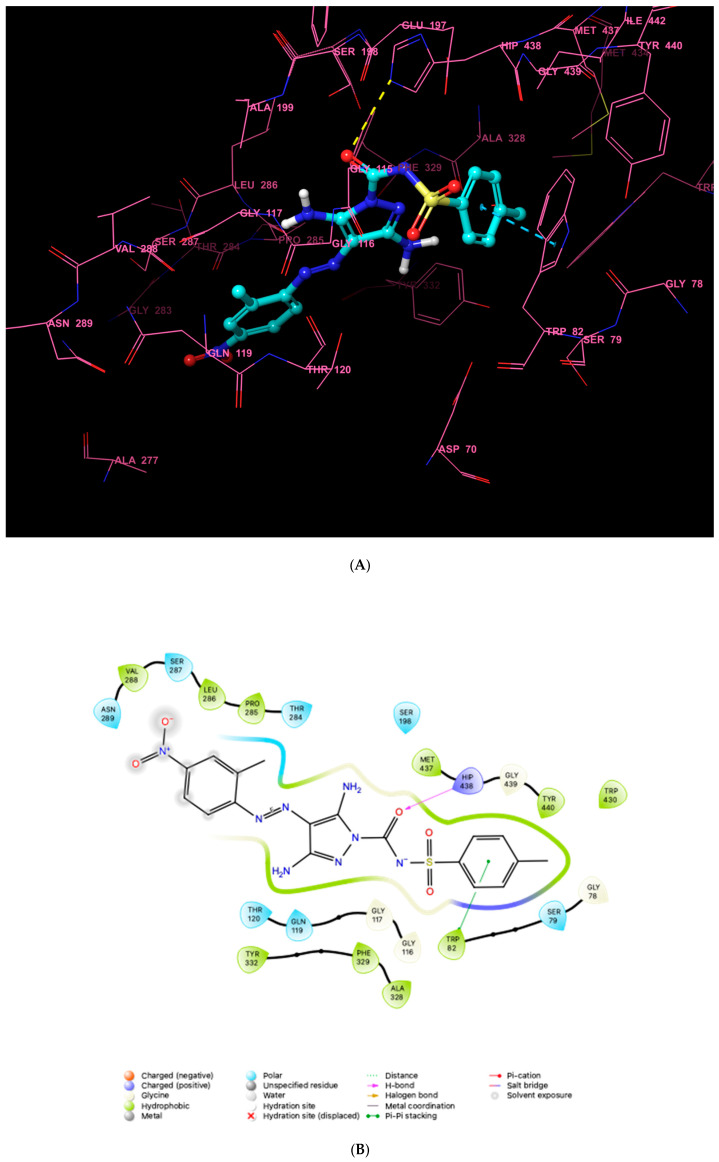
Docking pose of **5i** in BChE (PDB ID: 4BDS). (**A**) Predicted 3D binding orientation within the binding pocket (H-bonds: yellow dashed lines; π–π interactions: blue dashed lines). (**B**) 2D interaction diagram showing key contacts. Key residues were labelled for orientation, and the catalytic serine (Ser198) is emphasized in both panels.

For BChE, **5i** adopted a favorable pose within the broader and more permissive binding pocket, with a docking score of −3.89 kcal/mol. The predicted complex was supported by a combination of hydrophobic interactions and hydrogen bonding. Relative to AChE, the BChE site is more tolerant of diverse ligand shapes, which can allow alternative geometries and can help rationalize the strong BChE inhibition observed for selected members of the series (Figure 6 and Figure 7).

Overall, docking trends agreed with the experimental potency ranking. The two most active inhibitors, **5e** for AChE (*K*_I_ = 20.86 ± 1.61 nM) and **5i** for BChE (*K*_I_ = 31.21 ± 2.65 nM), also produced the most coherent interaction patterns, combining hydrogen bonding, aromatic contacts, and van der Waals complementarity in their respective binding environments. In AChE, ligands with more rigid aromatic character generally achieved better accommodation within the narrow gorge, whereas BChE more readily tolerated structural diversity, including carboxamide-containing motifs. Across the subset **5e**–**5i**, pose differences further indicated that steric demand and substituent electronics can redirect binding orientation; in some cases, more peripheral placements were favored, which may reduce interaction quality and docking performance. Collectively, these observations provide a structure-guided basis for subsequent optimization of this scaffold against both ChEs.

Taken together, the docking poses provide a structural rationale for the enzyme preference observed within this matched series. The AChE-leading profile of **5e** is consistent with efficient engagement of the aromatic gorge through Trp86-associated π–π anchoring together with a stabilizing Tyr124 hydrogen bond. In contrast, the BChE-leading behavior of **5i** is compatible with the larger and more permissive BChE pocket that can accommodate bulk and allow alternative hydrophobic/hydrogen-bonding arrangements. In this context, terminal aryl substitution appears to serve as a practical tuning handle, shifting binding orientation and complementarity between the two ChEs without altering the shared core scaffold.

### 2.4. Molecular Dynamics (MD) Simulation

To evaluate whether the top-ranked docking poses remain stable under explicit-solvent conditions, 100 ns MD simulations were performed for the 7XN1–**5e** and 4BDS–**5i** complexes in Cresset Flare (the full setup and parameters are described in Section 3.6). Complex stability and local flexibility were assessed using backbone RMSD and per-residue RMSF analyses.

**Figure 8:**
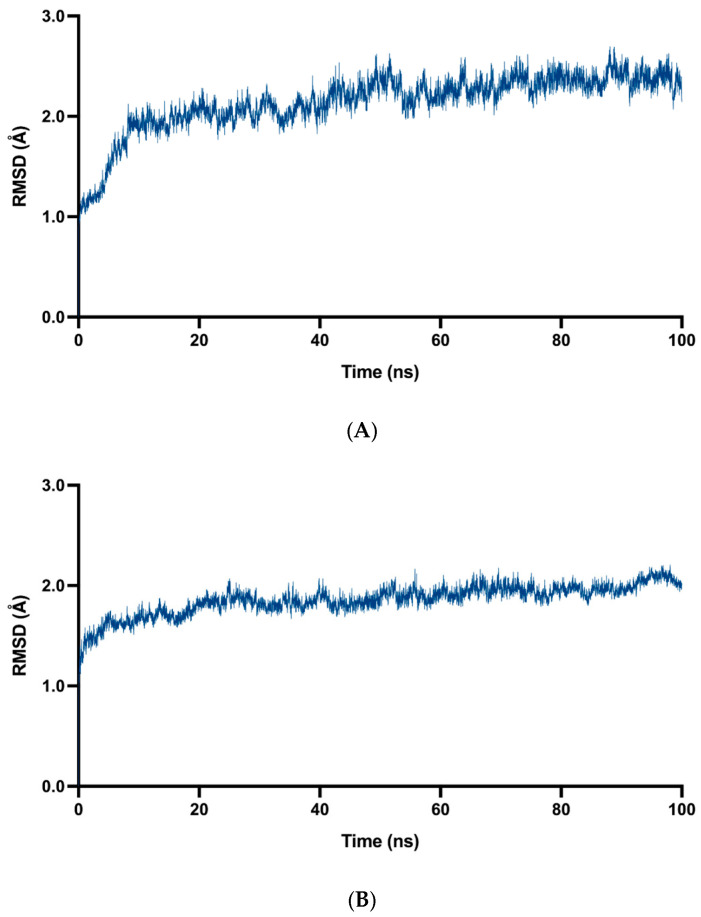
Backbone RMSD profiles of (**A**) 7XN1–**5e** and (**B**) 4BDS–**5i** over 100 ns MD.

**Figure 9:**
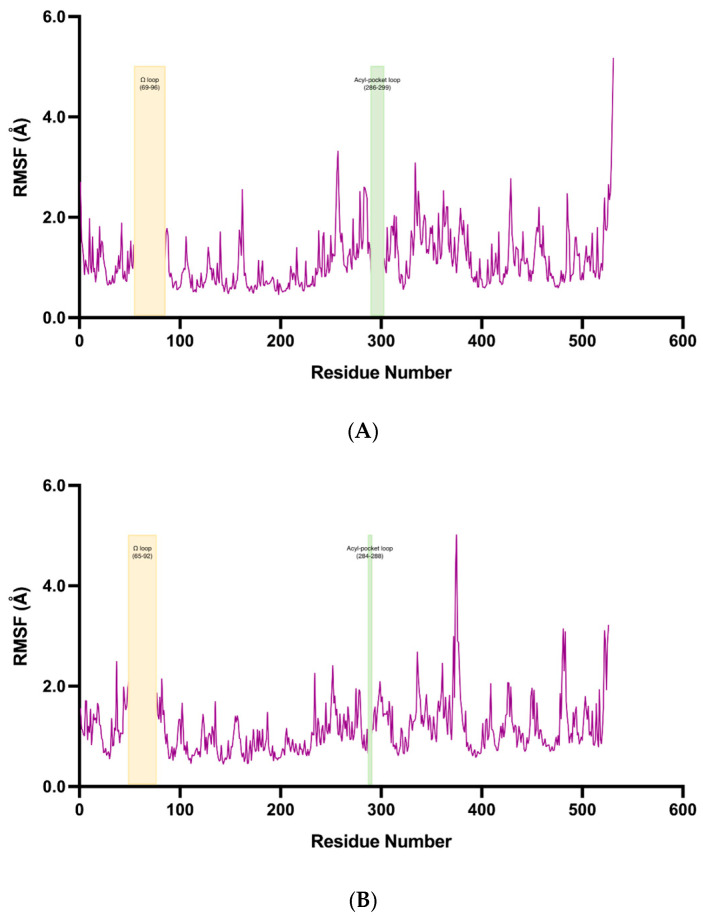
Per-residue RMSF profiles of (**A**) 7XN1–**5e** and (**B**) 4BDS–**5i** computed from the 100 ns trajectories. The Ω-loop and acyl-pocket loop regions are indicated on the plots (PDB residue numbering): AChE (7XN1) Ω-loop 69–96; acyl-pocket loop 286–299; BChE (4BDS) Ω-loop 65–92; acyl-pocket loop 284–288.

Backbone RMSD traces supported stable trajectories for both systems (Figure 8). When the post-equilibration interval was considered (10–100 ns), the 7XN1–**5e** complex showed a mean backbone RMSD of 2.21 ± 0.17 Å (range 1.73–2.70 Å), whereas 4BDS–**5i** exhibited a lower mean backbone RMSD of 1.89 ± 0.11 Å (range 1.57–2.21 Å). RMSF maxima were mainly confined to surface-exposed flexible regions. In particular, pronounced fluctuations were observed in the Ω-loop (AChE: Cys69–Cys96; BChE: Cys65–Cys92) and in the acyl-pocket loop region (annotated on Figure 9, PDB residue numbering), whereas the remainder of the fold remained comparatively rigid. The mean RMSF was 1.15 Å for 7XN1–**5e**, with a maximum of 5.17 Å at residue 531, and 1.17 Å for 4BDS–5i, with a maximum of 5.03 Å at residue 375. These patterns are consistent with preservation of the overall protein fold throughout the simulations.

Protein–ligand contact analysis (water excluded) further supported the docking-derived interaction models (Table 3). Where two occupancy values are listed for the same residue in Table 3, they correspond to distinct contact instances reported by Flare (e.g., alternative geometries or interaction definitions). In the 7XN1–**5e** trajectory, Trp86 displayed persistent π–π/aromatic contacts (62.9% and 53.1% frame occupancy). Additional stabilizing contributions included hydrogen-bond contacts involving Tyr124 (44.8%), Tyr449 (37.9%), Trp439 (36.0%), and Ser125 (32.0%), as well as a recurrent aromatic contact with Tyr449 (32.4%). In the 4BDS–**5i** trajectory, the complex was supported by hydrogen bonding to Ser287 (42.8% and 13.0%) and Gln119 (23.1%), along with an ionic interaction involving Asp70 (salt-bridge occupancy 26.6% and 15.1%) and a recurrent aromatic interaction with Tyr332 (32.0%). Overall, the persistence of these contacts for more than 100 ns indicates that the docking-derived binding modes are maintained during explicit-solvent sampling.

### 2.5. ADME/Tox. Study

In silico ADME/Tox. profiling was conducted for all ligands to obtain an initial perspective on pharmacokinetic behavior and potential developability liabilities (Table 4). Across the series, lipophilicity was moderate (M Log P 0.26–2.96), with the lowest values observed for **5e** and **5h** (0.26) and the highest for **5m** (2.96). As expected, higher lipophilicity may be compatible with membrane permeation, but it can also exacerbate aqueous solubility limitations. Consistent with this trade-off, most compounds were predicted to be poorly soluble (LogS < −4), which could negatively impact absorption. Within the set, **5e** showed comparatively better predicted solubility (LogS = −4.37) than more hydrophobic analogues such as **5m** (LogS = −5.11).

A notable outcome was the predicted lack of blood–brain barrier (BBB) permeability for all synthesized derivatives **5**(**a**–**m**), in contrast to THA. This profile suggests limited suitability for CNS exposure—undesirable for CNS-directed indications, but potentially advantageous when peripheral restriction is preferred. Regarding of metabolism-related liabilities, several compounds were predicted to inhibit CYP2C9, raising the risk of drug–drug interactions. In addition, **5b** and **5j** were predicted to inhibit CYP1A2 and CYP3A4, indicating a broader inhibition pattern, whereas **5e** showed a distinct profile with predicted inhibition of CYP2C19 and CYP3A4, pointing to compound-specific metabolic considerations.

Synthetic accessibility scores fell within a narrow band (3.53–3.83), consistent with moderate synthetic tractability and suggesting that optimization should remain feasible without a major increase in synthetic burden. Safety-related predictions were encouraging for selected endpoints: hepatotoxicity and neurotoxicity were predicted to be inactive for all compounds. However, nephrotoxicity was flagged for **5f** and **5g**, and carcinogenicity alerts were noted for **5e**, **5g**, **5h**, and **5i**, identifying liabilities that should be addressed during lead refinement. In addition, several derivatives (e.g., **5a**–**5d**, **5j**, **5l**, **5m**) were predicted as clinically toxic (active), supporting the need for cautious prioritization and follow-up experimental confirmation.

Drug-likeness analysis indicated that most ligands violated one or no Lipinski criteria, which can be consistent with oral drug-like space. In contrast, Ghose and Veber violations were observed across the series, suggesting that further adjustment of physicochemical space may be needed to improve overall developability. For comparison, THA displayed better predicted solubility (LogS = −3.27) and BBB permeability, but was predicted to inhibit multiple CYP enzymes and to show active neurotoxicity, highlighting the common exposure–safety trade-off in this target area.

Overall, the ADME/Tox. profile of the **5**(**a**–**m**) series appears favorable for specific organ-toxicity endpoints (inactive hepatotoxicity and neurotoxicity), supporting consideration for peripheral applications. At the same time, low solubility, CYP-inhibition liabilities, and carcinogenicity/nephrotoxicity alerts represent key hurdles for next-round design. Within the set, **5e** appears comparatively balanced physicochemically, but further structural refinement is still required to mitigate predicted toxicity-related liabilities.

## 3. Materials and Methods

### 3.1. Chemistry

Reagents and anhydrous solvents were obtained from commercial suppliers (Sigma-Aldrich, St. Louis, MO, USA; Merck, Darmstadt, Germany; Alfa Aesar, Haverhill, MA, USA; and TCI, Tokyo, Japan) and used without further purification. Melting points were determined in open capillaries on an SMP30 apparatus (Bibby Scientific/Stuart Equipment, Staffordshire, UK) and are reported uncorrected. FT-IR spectra were recorded on a PerkinElmer Spectrum 100 spectrometer. ^1^H and ^13^C NMR spectra were acquired on a Bruker Advance III 500 MHz instrument (Billerica, MA, USA) using DMSO-d_6_ and tetramethylsilane (TMS) as the internal standard; spectra were collected at 500 MHz (^1^H) and 125 MHz (^13^C). Reaction progress and product purity were monitored by TLC on Merck silica gel 60 F254 plates.

### 3.2. Comprehensive Synthetic Approaches for the Novel 4-Arylazo-3,5-Diamino-N-Tosyl-1H-Pyrazole-1-Carboxamide Derivatives ***5**(**a**–**m**)*

#### 3.2.1. Diazotization and Coupling

A primary aromatic amine (5 mmol) was dissolved in concentrated HCl (1.5 mL) and water (5 mL), and the mixture was cooled to 0–5 °C. A solution of NaNO_2_ (6 mmol in 5 mL water) was added dropwise over 15–20 min with continuous stirring. The mixture was maintained at 0–5 °C for a further 20 min to complete diazotization. The resulting diazonium solution was then added to malononitrile (5 mmol) dissolved in methanol (2 mL). The pH was adjusted to 6–7 using saturated sodium acetate, and the reaction mixture was stirred at 0–5 °C for 3 h, then kept overnight at room temperature in the dark. The precipitated solid was collected by filtration, washed several times with cold water, and recrystallized from ethanol to afford the hydrazone intermediate, which was used in the next step without additional purification.

#### 3.2.2. Cyclization to Pyrazoles

The diazo-containing malononitrile intermediate (2 mmol) was dissolved in ethanol (10 mL), and hydrazine monohydrate (3 mmol) was added. The mixture was stirred at room temperature for 2–3 h, then heated at 50 °C for ~1 h, with progress monitored by TLC. After completion, the solid product was filtered, washed with water, and used directly in the subsequent step without purification.

#### 3.2.3. Carbamoylation

The pyrazole intermediate (1 mmol) was dissolved in acetonitrile (5 mL), and 4-methylbenzenesulfonyl isocyanate (1.1 mmol) was added dropwise. The mixture was stirred at room temperature for 6–8 h until the reaction was complete (TLC). The resulting solid was filtered and dried under vacuum to yield the final products **5**(**a**–**m**), which were characterized by FT-IR, ^1^H NMR, ^13^C NMR, and melting point determination (Scheme 1). The products typically precipitated as intensely colored solids and were isolated by simple filtration; when needed, the crude materials were washed with diethyl ether to remove residual reagents and byproducts before final drying.

#### 3.2.4. 3,5-Diamino-4-(Phenyldiazenyl)-N-Tosyl-1H-Pyrazole-1-Carboxamide (**5a**)

Yield: 76.6%; Color: yellow solid; Melting Point: 206–207 °C; FT-IR (cm^−1^): 3429 (-NH_2_), 3331 (-NH_2_), 3221, 1703 (C=O), 1356, 1158 (symmetric) (S=O), 1088; H NMR (DMSO-d_6_, 500 MHz, δ ppm): 9.45 (s, 1H, -NH-), 7.93 (br.s, 2H, -NH_2_), 7.80 (d, *J* = 8.0 Hz, 2H, Ar-H), 7.53 (d, *J* = 8.5 Hz, 2H, Ar-H), 7.44 (t, *J* = 8.0 Hz, 1H, Ar-H), 7.30 (t, *J* = 7.5 Hz, 1H, Ar-H), 7.15 (d, *J* = 8.5 Hz, 2H, Ar-H), 6.17 (br.s, 2H, -NH_2_), 2.27 (s, 3H, -CH_3_); C NMR (DMSO-d_6_, 125 MHz, δ ppm): 151.42 (C=O), 150.58, 135.21, 133.42, 129.61, 129.35, 128.34, 121.45, 120.76, 113.87, 20.89 (-CH_3_).

#### 3.2.5. 3,5-Diamino-4-[(4-Chlorophenyl)Diazinyl]-N-Tosyl-1H-Pyrazole-1-Carboxamide (**5b**)

Yield: 73.2%; Color: light yellow solid; Melting Point: 295–296 °C; FT-IR (cm^−1^): 3462 (-NH_2_), 3421 (-NH_2_), 3349, 1712 (C=O), 1348, 1145 (symmetric) (S=O), 1088; H NMR (DMSO-d_6_, 500 MHz, δ ppm): 9.45 (s, 1H, -NH-), 7.97 (br.s, 2H, -NH_2_), 7.83 (d, *J* = 7.5 Hz, 2H, Ar-H), 7.52 (d, *J* = 8.5 Hz, 2H, Ar-H), 7.48 (d, *J* = 8.5 Hz, 2H, Ar-H), 7.15 (d, *J* = 7.5 Hz, 2H, Ar-H), 6.19 (br.s, 2H, -NH_2_), 2.27 (s, 3H, -CH_3_); C NMR (DMSO-d_6_, 125 MHz, δ ppm): 152.24 (C=O), 150.53, 135.17, 133.45, 132.26, 129.61, 129.35, 123.01, 120.78, 114.16, 20.89 (-CH_3_).

#### 3.2.6. 3,5-Diamino-4-[(4-Fluorophenyl)Diazinyl]-N-Tosyl-1H-Pyrazole-1-Carboxamide (**5c**)

Yield: 67.6%; Color: yellow solid; Melting Point: 215–216 °C; FT-IR (cm^−1^): 3466 (-NH_2_), 3423 (-NH_2_), 3349, 1704 (C=O), 1348, 1146 (symmetric) (S=O), 1090; H NMR (DMSO-d_6_, 500 MHz, δ ppm): 9.44 (s, 1H, -NH-), 7.92 (br.s, 2H, -NH_2_), 7.84–7.86 (m, 2H, Ar-H), 7.53 (d, *J* = 8.5 Hz, 2H, Ar-H), 7.27 (t, *J* = 9.0 Hz, 2H, Ar-H), 7.15 (d, *J* = 8.5 Hz, 2H, Ar-H), 6.15 (br.s, 2H, -NH_2_), 2.27 (s, 3H, -CH_3_); C NMR (DMSO-d_6_, 125 MHz, δ ppm): 163.08, 161.13, 150.56 (C=O), 150.24, 135.20, 133.42, 129.01, 123.24, 123.18, 120.75, 120.63, 116.21, 116.03, 113.72, 20.89 (-CH_3_).

#### 3.2.7. 3,5-Diamino-4-[(4-Methoxyphenyl)Diazinyl]-N-Tosyl-1H-Pyrazole-1-Carboxamide (**5d**)

Yield: 72.8%; Color: light yellow solid; Melting Point: 173–174 °C; FT-IR (cm^−1^): 3402 (-NH_2_), 3335 (-NH_2_), 3292, 1715 (C=O), 1294, 1147 (symmetric) (S=O), 1085; H NMR (DMSO-d_6_, 500 MHz, δ ppm): 9.42 (s, 1H, -NH-), 7.77 (d, *J* = 8.5 Hz, 4H, Ar-H and –NH_2_), 7.53 (d, *J* = 9.0 Hz, 2H, Ar-H), 7.14 (d, *J* = 8.5 Hz, 2H, Ar-H), 7.00 (d, *J* = 9.0 Hz, 2H, Ar-H), 6.09 (br.s, 2H, -NH_2_), 3.80 (s, 3H, -OCH_3_), 2.27 (s, 3H, -CH_3_); C NMR (DMSO-d_6_, 125 MHz, δ ppm): 159.79, 150.61 (C=O), 147.57, 135.25, 133.35, 129.60, 122.86, 120.70, 114.55, 113.17, 55.83 (-OCH_3_), 20.89 (-CH_3_).

#### 3.2.8. 3,5-Diamino-4-[(4-Nitrophenyl)Diazinyl]-N-Tosyl-1H-Pyrazole-1-Carboxamide (**5e**)

Yield: 65.2%; Color: dark red solid; Melting Point: 246–247 °C; FT-IR (cm^−1^): 3459 (-NH_2_), 3415 (-NH_2_), 3942, 1711 (C=O), 1335, 1146 (symmetric) (S=O), 1086; H NMR (DMSO-d_6_, 500 MHz, δ ppm): 9.49 (br.s, 1H, -NH-), 8.28 (d, *J* = 8.5 Hz, 2H, Ar-H), 8.20 (br.s, 1H, -NH-), 8.00 (d, *J* = 9.0 Hz, 2H, Ar-H), 7.53 (d, *J* = 8.5 Hz, 2H, Ar-H), 7.15 (d, *J* = 8.0 Hz, 2H, Ar-H), 6.57 (br.s, 1H, -NH-), 6.15 (br.s, 1H, -NH-), 2.27 (s, 3H, -CH_3_); C NMR (DMSO-d_6_, 125 MHz, δ ppm): 158.00 (C=O), 145.85, 135.08, 133.58, 129.62, 125.21, 120.88, 116.43, 20.90 (-CH_3_).

#### 3.2.9. 3,5-Diamino-4-[(4-Acetylphenyl)Diazinyl]-N-Tosyl-1H-Pyrazole-1-Carboxamide (**5f**)

Yield: 65.9%; Color: brown solid; Melting Point: 183–184 °C; FT-IR (cm^−1^): 3419 (-NH_2_), 3321 (-NH_2_), 3234, 1709 (C=O), 1677 (C=O), 1357, 1158 (symmetric) (S=O), 1087; H NMR (DMSO-d_6_, 500 MHz, δ ppm): 9.47 (s, 1H, -NH-), 8.01 (d, *J* = 8.5 Hz, 3H, Ar-H and –NH-), 7.90 (d, *J* = 8.5 Hz, 2H, Ar-H), 7.53 (d, *J* = 8.5 Hz, 2H, Ar-H), 7.15 (d, *J* = 8.0 Hz, 2H, Ar-H), 6.48 (br.s, 1H, -NH-), 6.10 (br.s, 1H, -NH-), 2.59 (s, 3H, -CH_3_), 2.27 (s, 3H, -CH_3_); C NMR (DMSO-d_6_, 125 MHz, δ ppm): 197.57 (Acetyl C=O), 156.51, 150.48 (C=O), 135.69, 135.13, 133.50, 129.76, 121.33, 120.82, 120.75, 115.32, 27.21 (Acetyl -CH_3_)., 20.90 (-CH_3_).

#### 3.2.10. 3,5-Diamino-4-[(4-Hydroxyphenyl)Diazinyl]-N-Tosyl-1H-Pyrazole-1-Carboxamide (**5g**)

Yield: 67.7%; Color: yellow solid; Melting Point: 216–217 °C; FT-IR (cm^−1^): 3449 (-NH_2_), 3328 (-NH_2_), 3241, 1697 (C=O), 1345, 1149 (symmetric) (S=O), 1085; H NMR (DMSO-d_6_, 500 MHz, δ ppm): 9.74 (s, 1H, -OH), 9.41 (s, 1H, -NH-), 7.70 (br.s, 2H, -NH_2_), 7.66 (d, *J* = 9.0 Hz, 2H, Ar-H), 7.52 (d, *J* = 8.5 Hz, 2H, Ar-H), 7.14 (d, *J* = 8.5 Hz, 2H, Ar-H), 6.81 (d, *J* = 9.0 Hz, 2H, Ar-H), 6.04 (br.s, 2H, -NH_2_), 2.27 (s, 3H, -CH_3_); C NMR (DMSO-d_6_, 125 MHz, δ ppm): 158.35, 150.63 (C=O), 146.48, 135.27, 133.33, 129.60, 123.02, 120.69, 115.87, 112.87, 20.89 (-CH_3_).

#### 3.2.11. 3,5-Diamino-4-[(3-Nitrophenyl)Diazinyl]-N-Tosyl-1H-Pyrazole-1-Carboxamide (**5h**)

Yield: 72.3%; Color: light orange solid; Melting Point: 214–215 °C; FT-IR (cm^−1^): 3459 (-NH_2_), 3417 (-NH_2_), 3339, 1709 (C=O), 1349, 1147 (symmetric) (S=O), 1080; H NMR (DMSO-d_6_, 500 MHz, δ ppm): 9.48 (s, 1H, -NH-), 8.08 (s, 1H, Ar-H), 7.85 (br.s, 2H, -NH_2_), 7.72 (d, *J* = 8.5 Hz, 2H, Ar-H), 7.58 (d, *J* = 8.0 Hz, 1H, Ar-H), 7.52 (d, *J* = 8.0 Hz, 1H, Ar-H), 7.15 (d, *J* = 8.5 Hz, 2H, Ar-H), 6.18 (br.s, 2H, -NH_2_), 2.28 (s, 3H, -CH_3_); C NMR (DMSO-d_6_, 125 MHz, δ ppm): 150.77 (C=O), 147.35, 135.32, 133.27, 129.68, 129.12, 123.49, 121.42, 120.71, 115.76, 114.52, 20.91 (-CH_3_).

#### 3.2.12. 3,5-Diamino-4-[(2-Methyl-4-Nitrophenyl)Diazinyl]-N-Tosyl-1H-Pyrazole-1-Carboxamide (**5i**)

Yield: 68.4%; Color: reddish solid; Melting Point: 219–220 °C; FT-IR (cm^−1^): 3415 (-NH_2_), 3355 (-NH_2_), 3329, 1698 (C=O), 1334, 1162 (symmetric) (S=O), 1088; H NMR (DMSO-d_6_, 500 MHz, δ ppm): 9.51 (s, 1H, -NH-), 8.40 (br.s, 1H, -NH-), 8.20 (s, 1H, Ar-H), 8.07 (d, *J* = 9.0 Hz, 1H, Ar-H), 7.98 (br.s, 1H, -NH-), 7.53 (d, *J* = 8.5 Hz, 2H, Ar-H), 7.15 (d, *J* = 8.5 Hz, 2H, Ar-H), 6.48 (br.s, 1H, -NH-), 6.17 (br.s, 1H, -NH-), 2.57 (s, 3H, -CH_3_), 2.27 (s, 3H, -CH_3_); C NMR (DMSO-d_6_, 125 MHz, δ ppm): 155.82, 150.33 (C=O), 145.54, 135.09, 133.56, 129.66, 129.60, 122.39, 120.87, 117.29, 114.46, 20.90 (-CH_3_), 18.15 (-CH_3_).

#### 3.2.13. 3,5-Diamino-4-[(3,4-Dichlorophenyl)Diazinyl]-N-Tosyl-1H-Pyrazole-1-Carboxamide (**5j**)

Yield: 79.6%; Color: yellow solid; Melting Point: 291–292 °C; FT-IR (cm^−1^): 3455 (-NH_2_), 3418 (-NH_2_), 3335, 1712 (C=O), 1350, 1147 (symmetric) (S=O), 1089; H NMR (DMSO-d_6_, 500 MHz, δ ppm): 9.45 (s, 1H, -NH-), 8.13 (s, 1H, Ar-H), 8.05 (br.s, 1H, -NH-), 7.79 (d, *J* = 8.5 Hz, 1H, Ar-H), 7.68 (d, *J* = 8.5 Hz, 1H, Ar-H), 7.52 (d, *J* = 9.0 Hz, 2H, Ar-H), 7.15 (d, *J* = 9.0 Hz, 2H, Ar-H), 6.44 (br.s, 1H, -NH-), 6.11 (br.s, 1H, -NH-), 2.27 (s, 3H, -CH_3_); C NMR (DMSO-d_6_, 125 MHz, δ ppm): 153.30, 150.46 (C=O), 135.14, 133.48, 132.33, 131.28, 129.61, 129.57, 121.73, 120.79, 114.67, 114.46, 20.89 (-CH_3_).

#### 3.2.14. 3,5-Diamino-4-[(3,4-Dimethoxyphenyl)Diazinyl]-N-Tosyl-1H-Pyrazole-1-Carboxamide (**5k**)

Yield: 85.2%; Color: light green solid; Melting Point: 169–170 °C; FT-IR (cm^−1^): 3422 (-NH_2_), 3330 (-NH_2_), 3234, 1709 (C=O), 1352, 1161 (symmetric) (S=O), 1088; H NMR (DMSO-d_6_, 500 MHz, δ ppm): 9.41 (s, 1H, -NH-), 7.80 (br.s, 2H, -NH_2_), 7.53 (d, *J* = 8.5 Hz, 2H, Ar-H), 7.50 (s, 1H, Ar-H), 7.36 (d, *J* = 8.0 Hz, 1H, Ar-H), 7.15 (d, *J* = 8.5 Hz, 2H, Ar-H), 7.02 (d, *J* = 8.0 Hz, 1H, Ar-H), 6.14 (br.s, 2H, -NH_2_), 3.83 (s, 3H, -OCH_3_), 3.80 (s, 3H, -OCH_3_), 2.27 (s, 3H, -CH_3_); C NMR (DMSO-d_6_, 125 MHz, δ ppm): 150.59 (C=O), 149.77, 149.63, 147.62, 135.26, 133.35, 129.62, 120.68, 117.10, 113.06, 111.62, 102.32, 56.08 (-CH_3_), 55.98 (-OCH_3_), 20.89 (-CH_3_).

#### 3.2.15. 3,5-Diamino-4-[(3,5-Dimethylphenyl)Diazinyl]-N-Tosyl-1H-Pyrazole-1-Carboxamide (**5l**)

Yield: 69.6%; Color: light yellow solid; Melting Point: 228–229 °C; FT-IR (cm^−1^): 3452 (-NH_2_), 3353 (-NH_2_), 3288, 1693 (C=O), 1338, 1153 (symmetric) (S=O), 1089; H NMR (DMSO-d_6_, 500 MHz, δ ppm): 9.43 (s, 1H, -NH-), 7.87 (br.s, 2H, -NH_2_), 7.53 (d, *J* = 8.5 Hz, 2H, Ar-H), 7.42 (s, 1H, Ar-H), Ar-H), 7.15 (d, *J* = 8.0 Hz, 2H, Ar-H), 6.94 (s, 1H, Ar-H), 6.14 (br.s, 2H, -NH_2_), 2.32 (s, 6H, -CH_3_), 2.27 (s, 3H, -CH_3_); C NMR (DMSO-d_6_, 125 MHz, δ ppm): 153.47 (C=O), 150.58, 138.37, 135.22, 133.40, 129.89, 129.61, 120.75, 119.30, 113.73, 21.43 (-CH_3_), 20.89 (-CH_3_).

#### 3.2.16. 3,5-Diamino-4-[(Perfluorophenyl)Diazinyl]-N-Tosyl-1H-Pyrazole-1-Carboxamide (**5m**)

Yield: 87.6%; Color: light yellow solid; Melting Point: 203–204 °C; FT-IR (cm^−1^): 3436 (-NH_2_), 3361 (-NH_2_), 3305, 1694 (C=O), 1359, 1165 (symmetric) (S=O), 1085; H NMR (DMSO-d_6_, 500 MHz, δ ppm): 9.50 (s, 1H, -NH-), 7.95 (br.s, 2H, -NH_2_), 7.58 (d, *J* = 8.5 Hz, 2H, Ar-H), 7.43 (d, *J* = 8.5 Hz, 2H, Ar-H), 6.28 (br.s, 2H, -NH_2_), 2.27 (s, 3H, -CH_3_); C NMR (DMSO-d_6_, 75 MHz, δ ppm): 159.43 (C=O), 147.32, 144.44, 142.31, 137.41, 130.00, 129.76, 127.95, 127.98, 126.09, 122.97, 122.19, 115.10, 20.92 (-CH_3_).

### 3.3. Cholinesterase Inhibition Assay

Cholinesterase inhibition by compounds **5**(**a**–**m**) was evaluated using a cuvette-based spectrophotometric assay adapted from Ellman’s method [58]. Assays were performed at pH 8.0 in 50 mM Tris–HCl containing 5 mM EDTA for AChE and 50 mM sodium phosphate buffer for BChE. AChE from Electrophorus electricus (Type V-S, ≥1000 U/mg; Sigma-Aldrich, Cat. No. C3389) and BChE from equine serum (≥900 U/mg; Sigma-Aldrich, Cat. No. C1057) were used. Acetylthiocholine iodide (ATChI; Sigma-Aldrich, Cat. No. A5751) and butrylthiocholine iodide (BTChI; Sigma-Aldrich, Cat. No. B3253) served as substrates, and 5,5′-dithiobis (2-nitrobenzoic acid) (DTNB; Sigma-Aldrich, Cat. No. D8130) was used as the chromogenic reagent. Inhibitor stock solutions were prepared in dimethyl sulfoxide (DMSO; Sigma-Aldrich, Cat. No. D8418) and added to achieve a final DMSO concentration was kept at 1% (*v*/*v*). Unless otherwise stated, all other chemicals and reagents were purchased from Sigma-Aldrich and used as received.

For each run, buffer (60 µL), inhibitor solution (10 µL), and enzyme (10 µL) were combined and pre-incubated at 37 °C for 10 min. Reactions were initiated by adding 10 µL of the appropriate substrate (ATChI for AChE or BTChI for BChE), followed immediately by 10 µL of DTNB to give a final DTNB concentration of 0.5 mM. Formation of the yellow 5-thio-2-nitrobenzoate anion was monitored at 412 nm [59]. Absorbance was recorded over 0–3 min with readings collected at 1-min intervals, and initial rates were expressed as ΔOD_412/min_. A solvent control (1% DMSO) was included in each run. In addition, a no-enzyme blank (all assay components except enzyme) was used to correct for background signal; where needed, compound-containing blanks were measured to account for any intrinsic absorbance and/or non-enzymatic contributions at 412 nm. A fixed pre-incubation time (10 min) was used throughout; time-dependent inhibition was not systematically evaluated and will be addressed in follow-up experiments using variable pre-incubation times and dilution-based reversibility tests.

Initial rates were measured at multiple substrate concentrations. For AChE, ATChI was varied at 0.080, 0.120, 0.160, 0.200, and 0.240 mM. For BChE, BTChI was varied at 0.050, 0.100, 0.150, 0.200, and 0.250 mM. At each substrate concentration, rates were determined in the absence and presence of increasing inhibitor concentrations. Lineweaver–Burk plots (1/v vs. 1/[S]) were constructed at each inhibitor level, and secondary replots of the slope (and where appropriate the intercept) versus inhibitor concentration were used to calculate *K*_I_ values [60,61]. Data are reported as mean ± SEM from three independent experiments. Representative Lineweaver–Burk plots and the corresponding secondary replots used for *K*_I_ estimation are shown in Figure 4 (additional plots are provided in the Supplementary Materials).

### 3.4. Cytotoxicity and Anticancer Assay

Beas-2B (ATCC CRL-9609), A549 (ATCC CCL-185), and MCF-7 (ATCC HTB-22) cell lines were obtained from ATCC (Manassas, VA, USA). Cells were cultured in DMEM containing 2 mM L-glutamine, 10% heat-inactivated FBS, and 1% penicillin–streptomycin, and maintained at 37 °C under 5% CO_2_ (≈95% relative humidity). Cultures were passaged at ~80% confluence, and morphology/confluence were checked by inverted phase-contrast microscopy.

Cytotoxicity of compounds **5**(**a**–**m**) was evaluated using the MTT (3-(4, 5-dimethylthiazolyl-2)-2, 5-diphenyltetrazolium bromide) (Mosmann) assay with minor modifications [62]. Cells were seeded in 96-well flat-bottom plates at 5 × 10^3^ cells/well in 100 µL and allowed to attach for 24 h. Compounds were then applied at 10–160 µM (10, 20, 40, 80, and 160 µM) for 24 h. The medium was removed and replaced with 100 µL/well MTT solution (1 mg/mL in PBS; Serva, CAS No. 298-93-1). After 4 h at 37 °C, formazan was dissolved in DMSO (100 µL/well) and absorbance was recorded at 550 nm. Viability was calculated relative to untreated controls (100%). 5-FU served as the reference drug. IC_50_ values were obtained from dose–response curves using CalcuSyn v2.1 (Biosoft, Cambridge, UK), with all conditions tested in triplicate [63].

### 3.5. Molecular Docking and ADME/Tox Analysis

Molecular docking was performed in the Schrödinger Small-Molecule Drug Discovery Suite (Mac-compatible release 2025-1). Crystal structures of AChE and BChE were downloaded from the RCSB Protein Data Bank (PDB IDs: 7XN1 [56] and 4BDS [57]) and prepared with the Protein Preparation Wizard. Preparation included hydrogen addition, removal of crystallographic water molecules, and optimization of ionization states under physiological pH conditions. The selected PDB entries correspond to human AChE (7XN1) and human BChE (4BDS); the potential impact of using non-human enzymes in the in vitro inhibition assays is acknowledged as a study limitation.

Ligands **5**(**a**–**m**) were drawn in ChemDraw v21 (PerkinElmer, Waltham, MA, USA) and processed in LigPrep to generate minimized 3D structures. OPLS4 was used for ligand minimization, and Epik was applied at pH 7.0 to generate relevant protonation/tautomeric states [64]. Binding-site features were examined with SiteMap [65], and docking grids were defined in Receptor Grid Generation around the corresponding enzyme pockets. Docking was run with Glide using the extra precision (XP) protocol [66,67].

In parallel, predicted ADME descriptors for compounds **5**(**a**–**m**) were obtained from the SwissADME web platform [68] to provide an initial developability-oriented profile.

### 3.6. Molecular Dynamics (MD) Analysis

MD simulations were carried out in Cresset Flare (Litlington, UK) to probe the stability of the docked poses for 7XN1–**5e** and 4BDS–**5i** [69]. Proteins were parameterized with an AMBER protein force field. Ligands used GAFF2 parameters, and AM1-BCC charges were assigned. Each complex was solvated in explicit TIP3P water (truncated octahedron box; 10 Å buffer). The ionic strength was set to 0.150 M NaCl, and counterions were added to neutralize the net charge. All simulations were based on the same human crystal structures used for docking (AChE: 7XN1; BChE: 4BDS).

Systems were energy-minimized in Flare, including a pre-minimization step to a tolerance of 0.25 kcal·mol^−1^·Å^−1^. Production simulations were run in the NPT ensemble (298 K, 1.0 bar) with particle mesh Ewald (PME) electrostatics and a 10 Å non-bonded cutoff. A 4 fs timestep was used with hydrogen mass repartitioning (factor 1.50). Each system underwent Flare’s seven-stage equilibration, followed by a 100 ns production run on a GPU platform.

Trajectory analysis was performed in Flare. Backbone RMSD and per-residue RMSF were exported as “Plot Data” tables. RMSD statistics reported in the Results Section 2 were calculated over 10–100 ns. Protein–ligand contact persistence was obtained from Flare contact analysis and reported as % of frames in which a contact was present (water-mediated contacts excluded).

## 4. Conclusions

In this study, we synthesized thirteen new 4-arylazo-3,5-diamino-*N*-tosyl-1*H*-pyrazole-1-carboxamide derivatives **5**(**a**–**m**) and evaluated them as inhibitors of AChE and BChE. Overall, all compounds showed strong inhibition of both enzymes, and several analogues were more potent than the reference inhibitor THA under our assay conditions. The most active AChE inhibitor was the 4-nitro derivative **5e** (*K*_I_ = 20.86 ± 1.61 nM), which was substantially stronger than THA (*K*_I_ = 164.40 ± 20.84 nM). Computational analyses helped explain these experimental findings. Docking suggested reasonable binding poses driven by a balance of polar and hydrophobic contacts within the active-site gorge/pocket. These binding hypotheses were further supported by explicit-solvent molecular dynamics simulations: the selected complexes (7XN1–**5e** and 4BDS–**5i**) remained stable over 100 ns, and the key interaction patterns observed during the simulations were consistent with the docking models (for example, persistent aromatic contacts involving Trp86 in the 7XN1–**5e** system). We also examined developability-related properties using in silico ADME/Tox predictions. The compounds generally showed moderate lipophilicity but low predicted aqueous solubility, and none were predicted to be blood–brain barrier permeant. This may limit their suitability for central nervous system indications unless optimized, but it could be compatible with peripheral applications. In addition, some derivatives were flagged for potential liabilities (e.g., predicted nephrotoxicity for **5f**/**5g** and carcinogenicity alerts for **5e**/**5g**/**5h**/**5i**), indicating that further optimization should focus not only on potency but also on safety-related properties. Taken together, the 4-arylazo-3,5-diamino-*N*-tosyl-1*H*-pyrazole-1-carboxamide scaffold represents a strong starting point for ChEI development. Future work should prioritize improving solubility, addressing predicted toxicity/metabolic liabilities, and fine-tuning terminal aryl substitution patterns to broaden the therapeutic window, guided by the structure-based interaction features identified here.

## Figures and Tables

**Table 1:** In vitro inhibition constants (*K*_I_, nM) of compounds **5**(**a**–**m**) against AChE and BChE, with tacrine (THA) as the reference inhibitor.

Compound ID	AChE		BChE	
	*K*_I_ (nM)	*R* ^2^	*K*_I_ (nM)	*R* ^2^
**5a**	30.65 ± 2.54	0.9874	43.78 ± 2.68	0.9876
**5b**	30.41 ± 2.65	0.9864	35.58 ± 2.06	0.9881
**5c**	42.48 ± 3.36	0.9884	31.45 ± 2.80	0.9886
**5d**	29.84 ± 2.37	0.9875	57.32 ± 3.65	0.9870
**5e**	20.86 ± 1.61	0.9883	87.07 ± 5.67	0.9868
**5f**	29.99 ± 2.30	0.9885	86.18 ± 4.92	0.9888
**5g**	46.07 ± 2.31	0.9892	44.56 ± 3.94	0.9882
**5h**	28.78 ± 2.28	0.9873	62.41 ± 6.11	0.9867
**5i**	38.37 ± 2.05	0.9875	31.21 ± 2.65	0.9890
**5j**	36.52 ± 2.71	0.9886	34.13 ± 2.99	0.9888
**5k**	27.04 ± 2.10	0.9879	34.55 ± 3.21	0.9872
**5l**	32.82 ± 2.44	0.9888	35.70 ± 3.08	0.9889
**5m**	27.20 ± 2.20	0.9876	53.05 ± 3.31	0.9874
**THA** ^a^	164.40 ± 20.84	0.9716	341.80 ± 53.35	0.9568

^a^ Tacrine.

**Table 2:** Cytotoxicity (IC_50_, µM) of compounds **5**(**a**–**m**) in Beas-2B, A549, and MCF-7 cells and corresponding selectivity indices (*S*_I_); 5-FU was used as the reference drug. *S*_I_ was calculated as IC_50_(Beas-2B)/IC_50_(cancer).

Compound ID	IC_50_ (μM)			*S*_I_	
	Beas-2B	A549	MCF-7	A549	MCF-7
**5a**	185.12	226.99	ND ^b^	0.82	ND ^b^
**5b**	221.49	260.00	142.55	0.85	1.55
**5c**	502.47	139.53	113.76	3.60	4.42
**5d**	10.36	105.34	28.18	0.10	0.37
**5e**	93.61	236.01	1245.98	0.40	0.08
**5f**	496.79	492.63	258.05	1.01	1.93
**5g**	28.28	26.98	129.93	1.05	0.22
**5h**	88.38	110.27	ND ^b^	0.80	ND ^b^
**5i**	272.25	82.96	ND ^b^	3.28	ND ^b^
**5j**	ND ^a^	212.18	ND ^b^	ND ^b^	ND ^b^
**5k**	62.18	114.30	271.94	0.54	0.23
**5l**	200.00	174.52	277.07	1.15	0.72
**5m**	226.06	ND ^a^	ND ^b^	ND ^b^	ND ^b^
**5-FU** ^a^	213.26	249.08	248.79	0.86	ND ^b^

^a^ 5-Fluorouracil. ^b^ Not determined.

**Table 3:** Persistent protein–ligand interactions observed during 100 ns MD simulations (Cresset Flare).

Complex	Residue	Interaction Type	Occupancy (% Frames)
7XN1–**5e**	Trp86	π–π/aromatic contact	62.9; 53.1
7XN1–**5e**	Tyr124	Hydrogen bond	44.8
7XN1–**5e**	Tyr449	Hydrogen bond	37.9
7XN1–**5e**	Trp439	Hydrogen bond	36.0
7XN1–**5e**	Tyr449	π–π/aromatic contact	32.4
7XN1–**5e**	Ser125	Hydrogen bond	32.0
4BDS–**5i**	Ser287	Hydrogen bond	42.8; 13.0
4BDS–**5i**	Tyr332	π–π/aromatic contact	32.0
4BDS–**5i**	Asp70	Salt bridge/ionic interaction	26.6; 15.1
4BDS–**5i**	Gln119	Hydrogen bond	23.1

**Table 4:** Predicted ADME/Tox. properties of compounds **5**(**a**–**m**) and tacrine (THA), including lipophilicity (M Log P), aqueous solubility (LogS), BBB permeability, CYP inhibition flags, synthetic accessibility score, and selected toxicity alerts (hepatotoxicity, neurotoxicity, nephrotoxicity, and carcinogenicity).

Properties	Parameters	Compound ID													
		5a	5b	5c	5d	5e	5f	5g	5h	5i	5j	5k	5l	5m	THA ^a^
Physicochemical properties	MW (g/mol)	399.43	433.87	417.42	429.45	444.42	441.46	415.43	444.42	458.45	468.32	459.48	427.48	489.38	198.26
	Heavy atoms	28	29	29	30	31	31	29	31	32	30	32	30	33	15
	Aromatic heavy atoms	17	17	17	17	17	17	17	17	17	17	17	17	17	10
	Rotatable atoms	6	6	6	7	7	7	6	7	7	6	8	6	6	0
	H-bond acceptors	6	6	7	7	8	7	7	8	8	6	8	6	11	1
	H-bond donors	3	3	3	3	3	3	4	3	3	3	3	3	3	1
	Molar refractivity	103.89	108.90	103.85	110.38	112.71	114.08	105.91	112.71	117.68	113.91	116.87	113.82	103.68	63.58
Lipophilicity	M Log P	1.04	1.54	1.42	0.78	0.26	0.68	0.54	0.26	0.49	2.04	0.53	1.51	2.96	2.33
Water solubility	LogS (ESOL)	−4.30	−4.90	−4.46	−4.38	−4.37	−4.25	−4.16	−4.37	−4.67	−5.49	−4.45	−4.91	−5.11	−3.27
Pharmacokinetics	BBB permeant	No	No	No	No	No	No	No	No	No	No	No	No	No	Yes
	CYP1A2 inhibitor	No	No	Yes	No	No	No	No	No	No	Yes	No	No	Yes	Yes
	CYP2C19 inhibitor	No	No	No	No	Yes	No	No	Yes	Yes	Yes	Yes	No	No	No
	CYP2C9 inhibitor	Yes	Yes	Yes	Yes	Yes	Yes	No	Yes	Yes	Yes	Yes	Yes	No	No
	CYP3A4 inhibitor	No	Yes	No	Yes	Yes	Yes	No	No	Yes	Yes	Yes	Yes	No	Yes
Druglikeness	Lipinski violation	0	0	0	1	1	1	1	1	1	0	1	0	0	0
	Ghose violation	Yes	Yes	Yes	Yes	Yes	Yes	Yes	Yes	Yes	Yes	Yes	Yes	Yes	Yes
	Veber violation	1	1	1	1	1	1	1	1	1	1	1	1	1	1
Medicinal chemistry	Synthetic accessibility	3.57	3.53	3.56	3.65	3.67	3.67	3.58	3.71	3.80	3.55	3.83	3.79	3.59	2.08
Organ toxicity	Hepatotoxicity	Inactive	Inactive	Inactive	Inactive	Inactive	Inactive	Inactive	Inactive	Inactive	Inactive	Inactive	Inactive	Inactive	Inactive
	Neurotoxicity	Inactive	Inactive	Inactive	Inactive	Inactive	Inactive	Inactive	Inactive	Inactive	Inactive	Inactive	Inactive	Inactive	Active
	Nephrotoxicity	Inactive	Inactive	Inactive	Inactive	Inactive	Active	Active	Inactive	Inactive	Inactive	Inactive	Inactive	Inactive	Inactive
Toxicity endpoints	Carcinogenicity	Inactive	Inactive	Inactive	Inactive	Active	Inactive	Active	Active	Active	Inactive	Inactive	Inactive	Inactive	Inactive
	BBB-barrier	Active	Active	Active	Inactive	Active	Active	Inactive	Active	Active	Active	Inactive	Active	Active	Active
	Clinical toxicity	Active	Active	Active	Active	Inactive	Inactive	Active	Inactive	Inactive	Active	Inactive	Active	Active	Active

^a^ Tacrine.

## Data Availability

The data presented in this study are contained within the article and the Supplementary Materials.

## Supplementary Materials

The following supporting information can be downloaded at: https://www.mdpi.com/article/10.3390/ph19020239/s1, Figure S1: H NMR spectrum of compound 5a (500 MHz, in DMSO-d_6_); Figure S2: C NMR spectrum of compound 5a (125 MHz, in DMSO-d_6_); Figure S3: H NMR spectrum of compound 5b (500 MHz, in DMSO-d_6_); Figure S4: C NMR spectrum of compound 5b (125 MHz, in DMSO-d_6_); Figure S5: H NMR spectrum of compound 5c (500 MHz, in DMSO-d_6_); Figure S6: C NMR spectrum of compound 5c (125 MHz, in DMSO-d_6_); Figure S7: H NMR spectrum of compound 5d (500 MHz, in DMSO-d_6_); Figure S8: C NMR spectrum of compound 5d (125 MHz, in DMSO-d_6_); Figure S9: H NMR spectrum of compound 5d (500 MHz, in DMSO-d_6_); Figure S10: C NMR spectrum of compound 5e (125 MHz, in DMSO-d_6_); Figure S11: H NMR spectrum of compound 5f (500 MHz, in DMSO-d_6_); Figure S12: C NMR spectrum of compound 5f (125 MHz, in DMSO-d_6_); Figure S13: H NMR spectrum of compound 5g (500 MHz, in DMSO-d_6_); Figure S14: C NMR spectrum of compound 5g (125 MHz, in DMSO-d_6_); Figure S15: H NMR spectrum of compound 5i (500 MHz, in DMSO-d_6_); Figure S16: C NMR spectrum of compound 5i (125 MHz, in DMSO-d_6_); Figure S17: H NMR spectrum of compound 5j (500 MHz, in DMSO-d_6_); Figure S18: C NMR spectrum of compound 5j (125 MHz, in DMSO-d_6_); Figure S19: H NMR spectrum of compound 5k (500 MHz, in DMSO-d_6_); Figure S20: C NMR spectrum of compound 5k (125 MHz, in DMSO-d_6_); Figure S21: H NMR spectrum of compound 5l (500 MHz, in DMSO-d_6_); Figure S22: C NMR spectrum of compound 5l (125 MHz, in DMSO-d_6_).
