# Supplementary material for: Design and Synthesis of 4-Arylazo Pyrazole Carboxamides as Dual AChE/BChE Inhibitors: Kinetic and In Silico Evaluation
Source: Pharmaceuticals (Basel). 2026 Jan 29;19(2):239. doi: 10.3390/ph19020239 (PMC12943507; doi:10.3390/ph19020239)
Supplement: Supplementary file 1 [file pharmaceuticals-19-00239-s001.zip › pharmaceuticals-4077767-supplementary.pdf]

Supplementary Materials

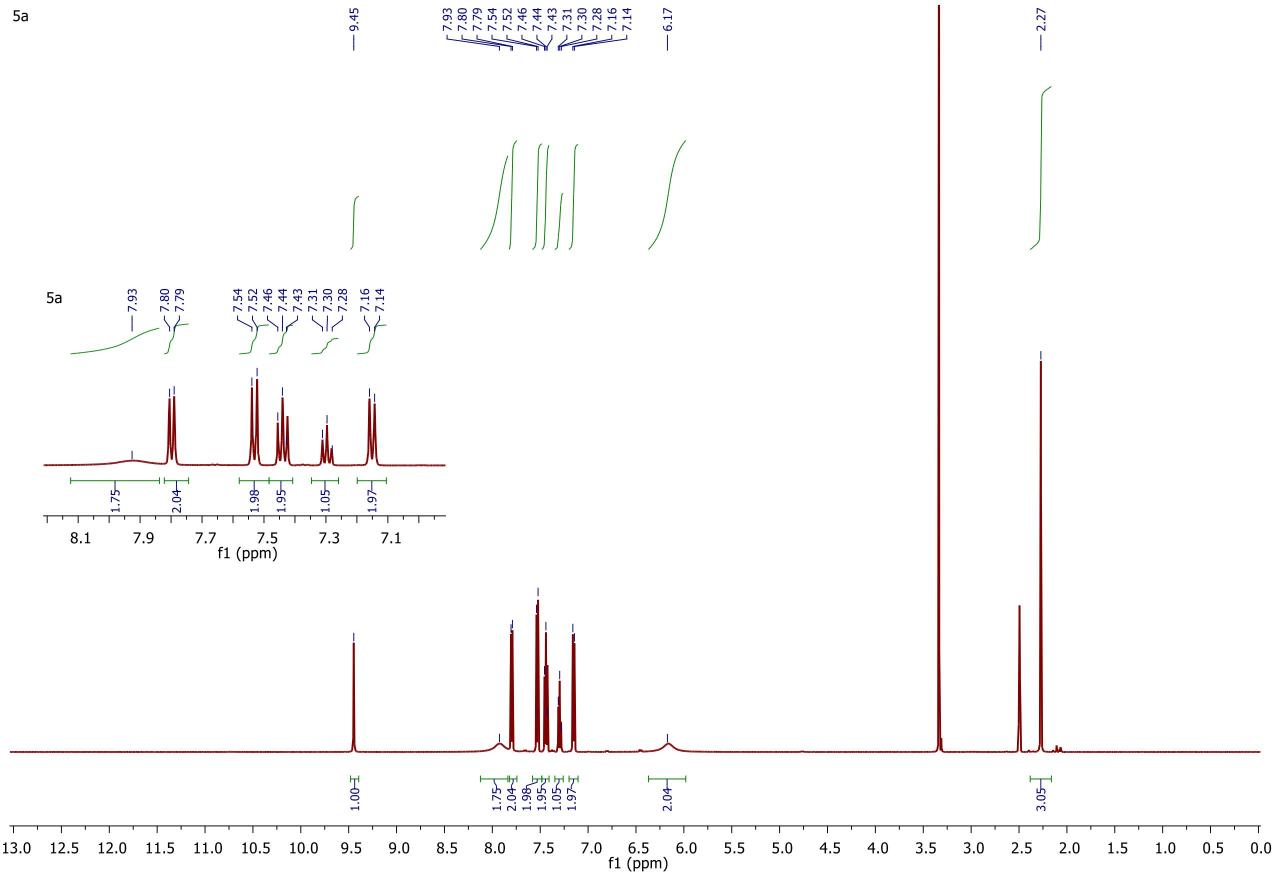

Figure S1: H NMR spectrum of compound **5a** (500 MHz, in DMSO-d$_6$).

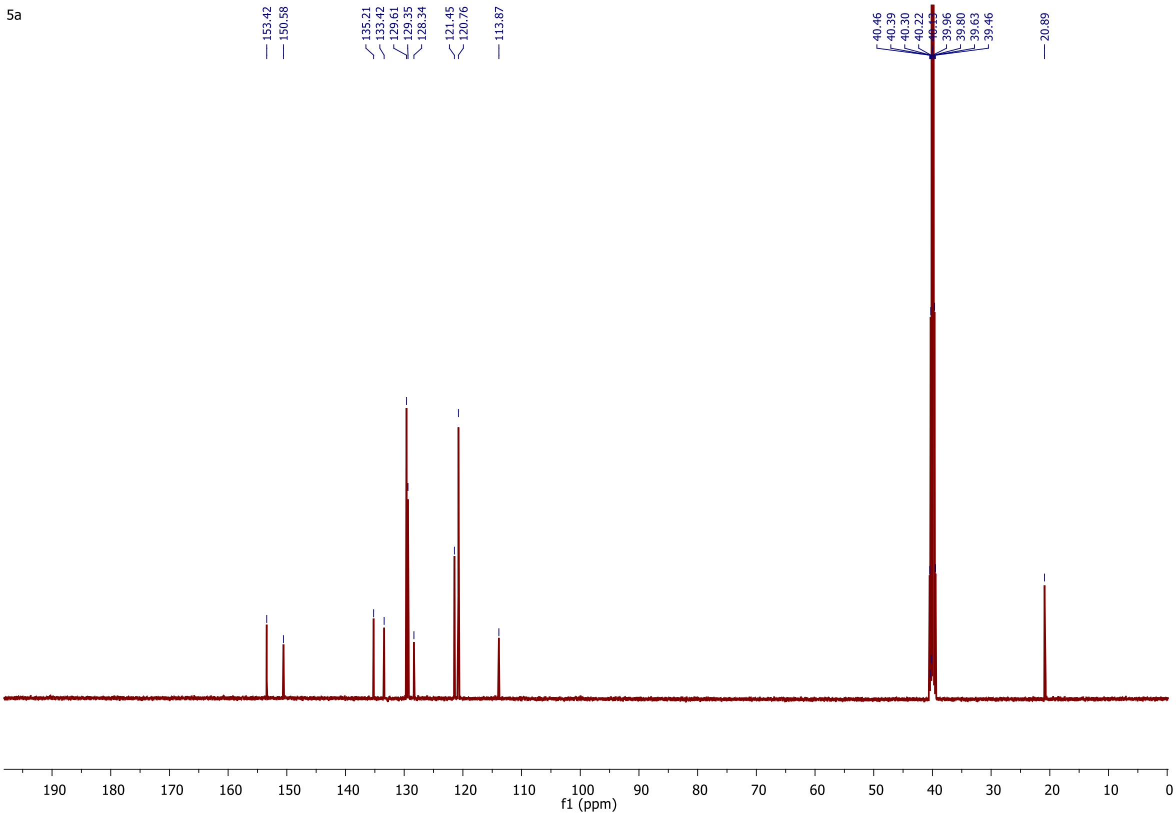

Figure S2: C NMR spectrum of compound **5a** (125 MHz, in DMSO-d_6).

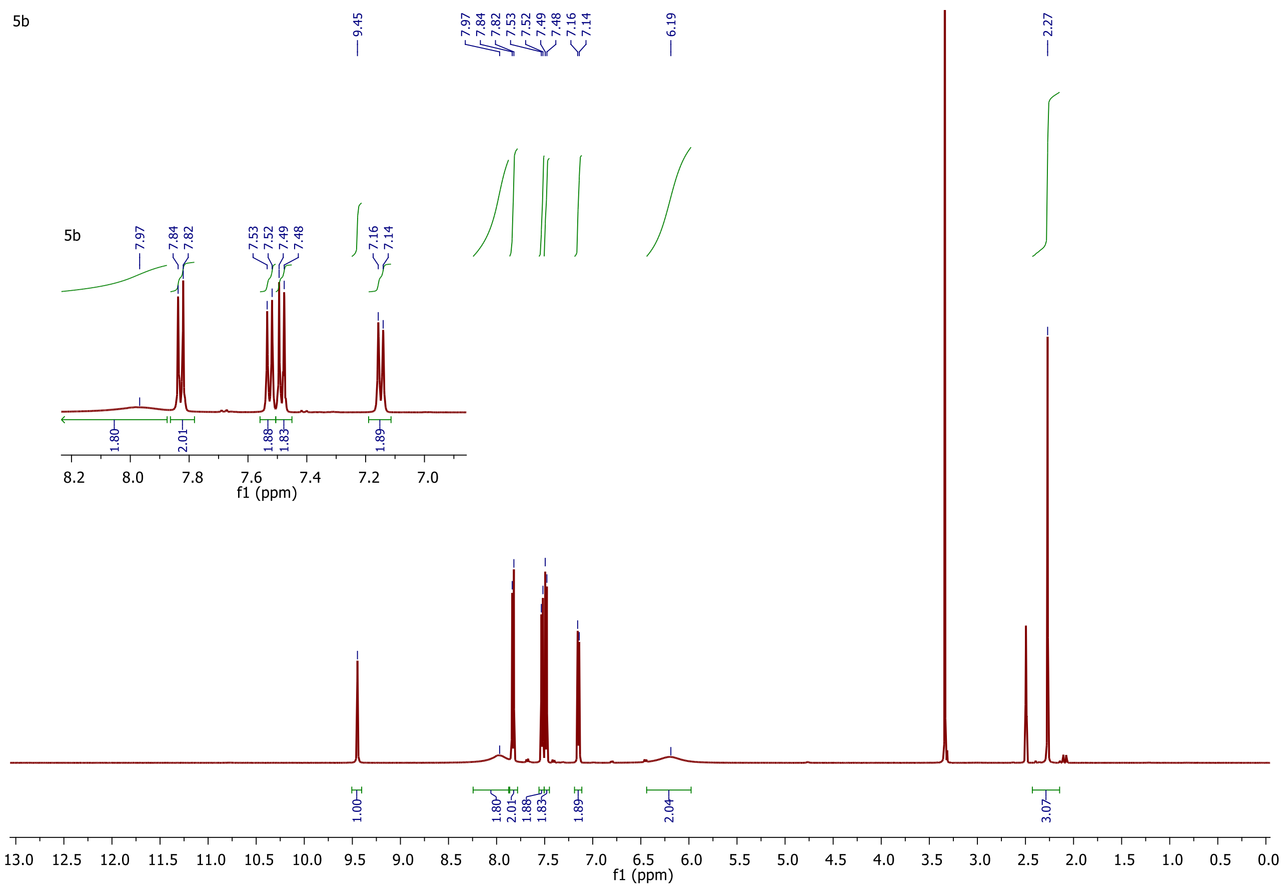

Figure S3: H NMR spectrum of compound **5b** (500 MHz, in DMSO-d_6).

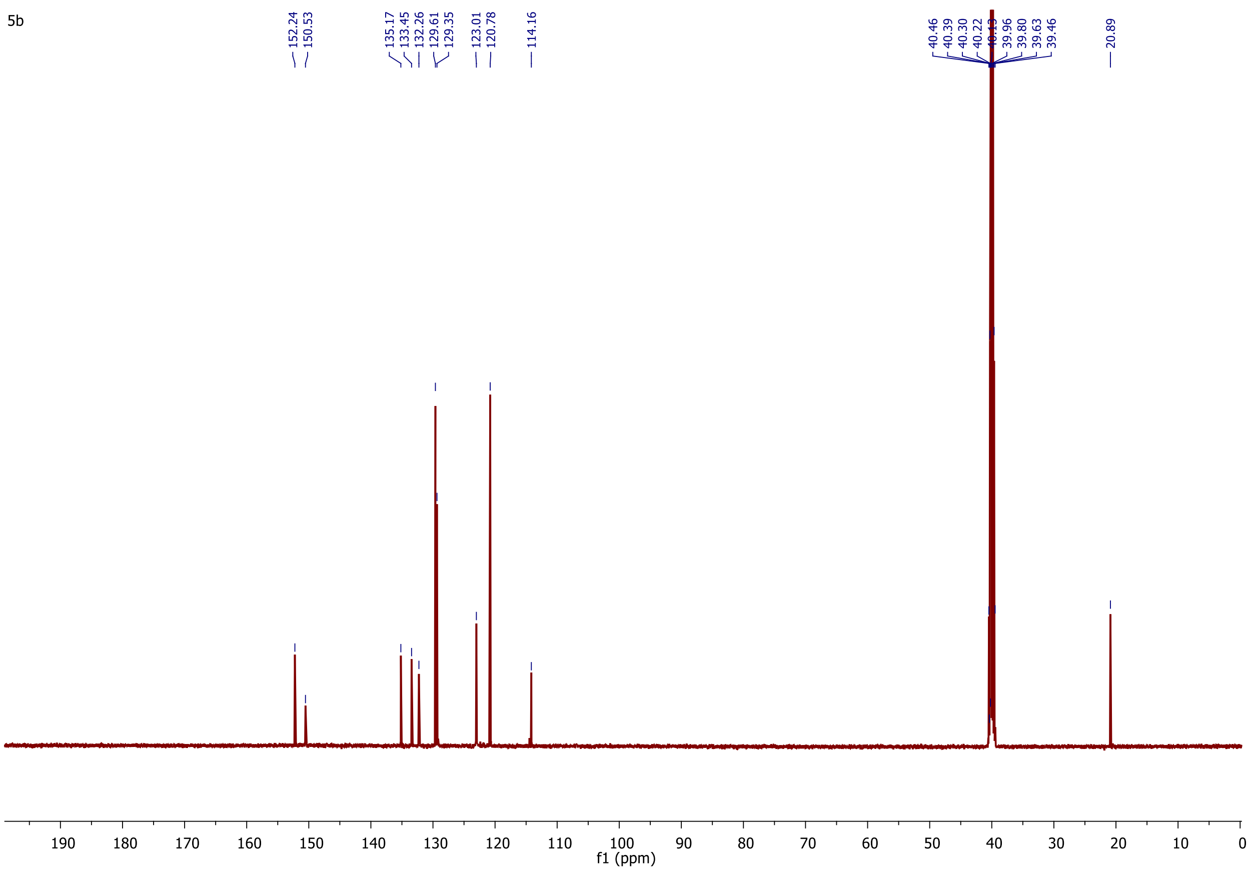

Figure S4: C NMR spectrum of compound **5b** (125 MHz, in DMSO-d_6).

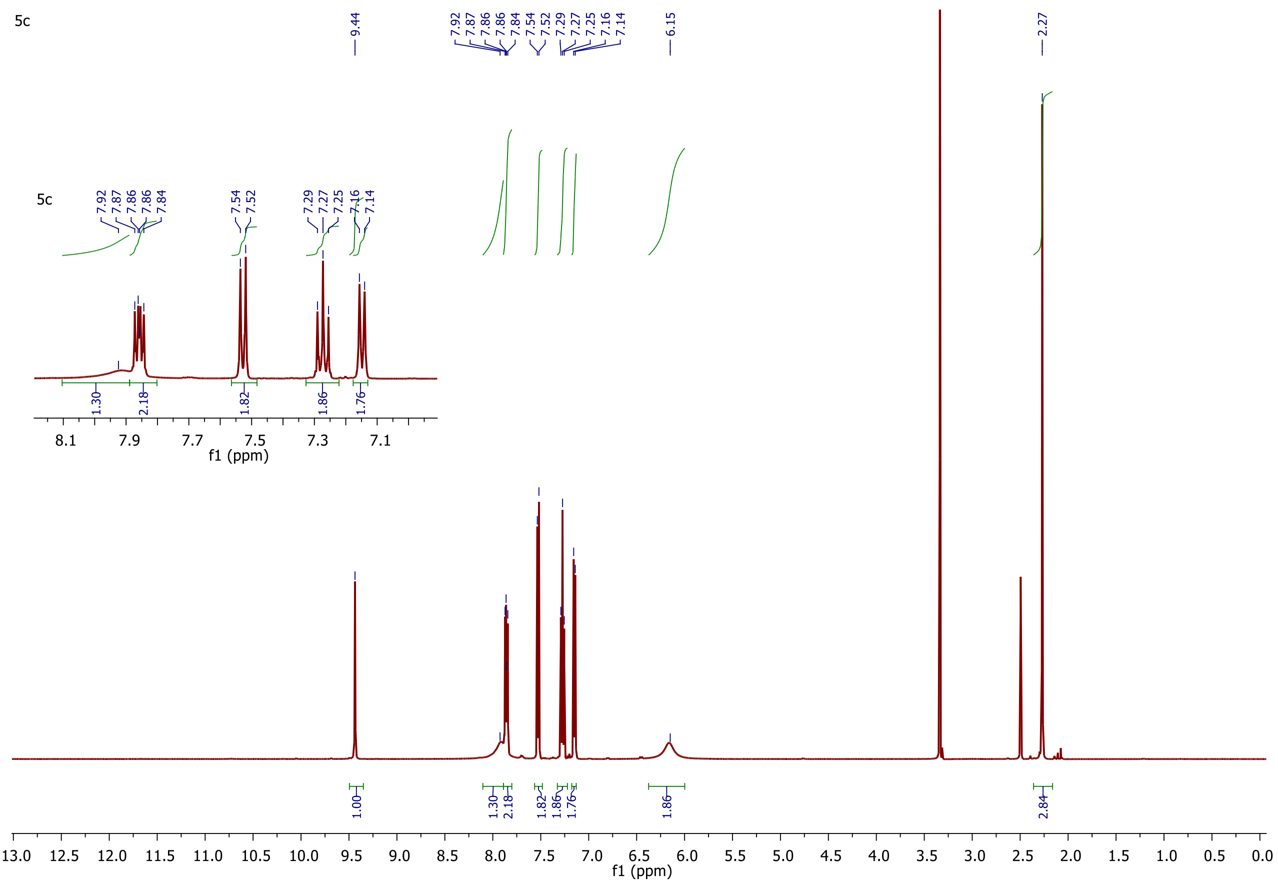

Figure S5: H NMR spectrum of compound **5c** (500 MHz, in DMSO-d_6).

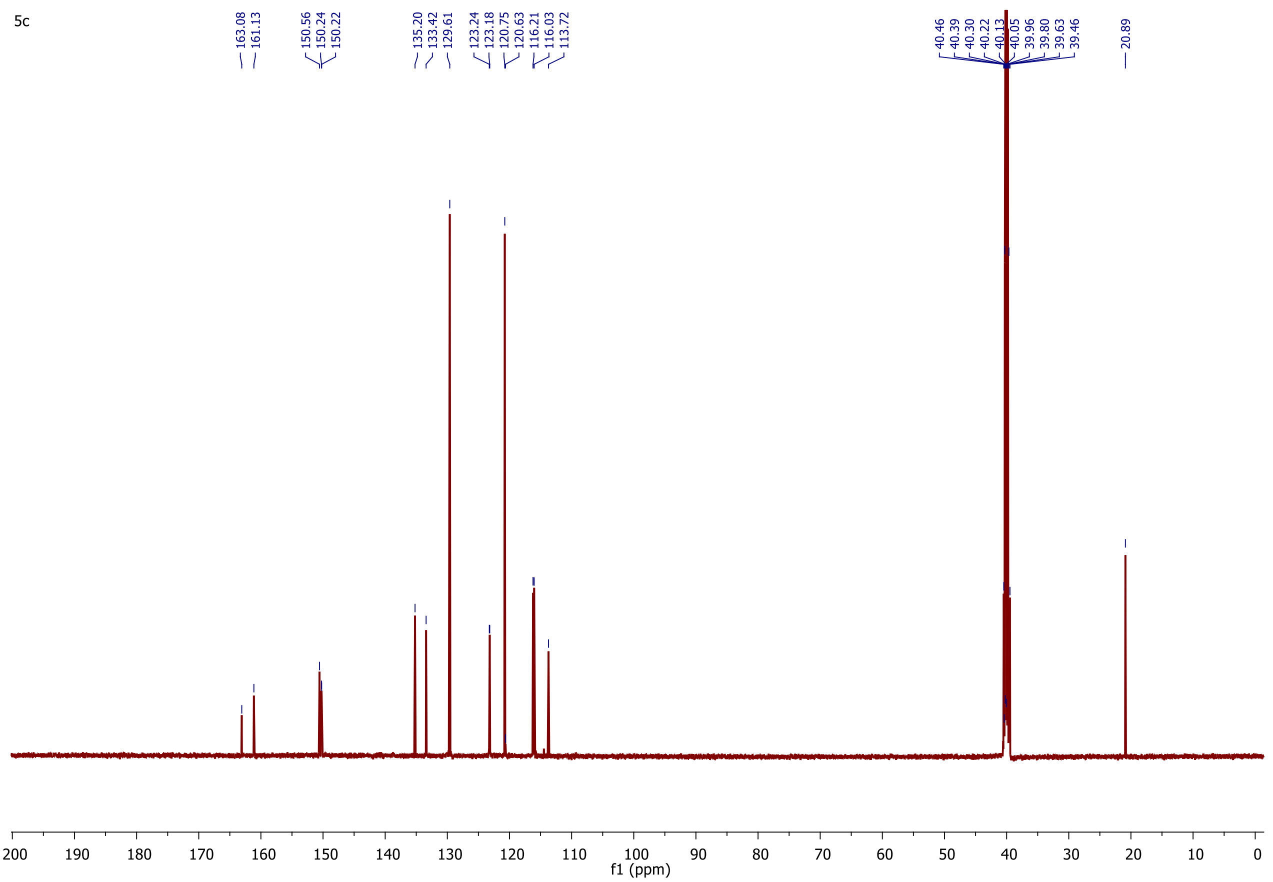

Figure S6: C NMR spectrum of compound **5c** (125 MHz, in DMSO-d_6).

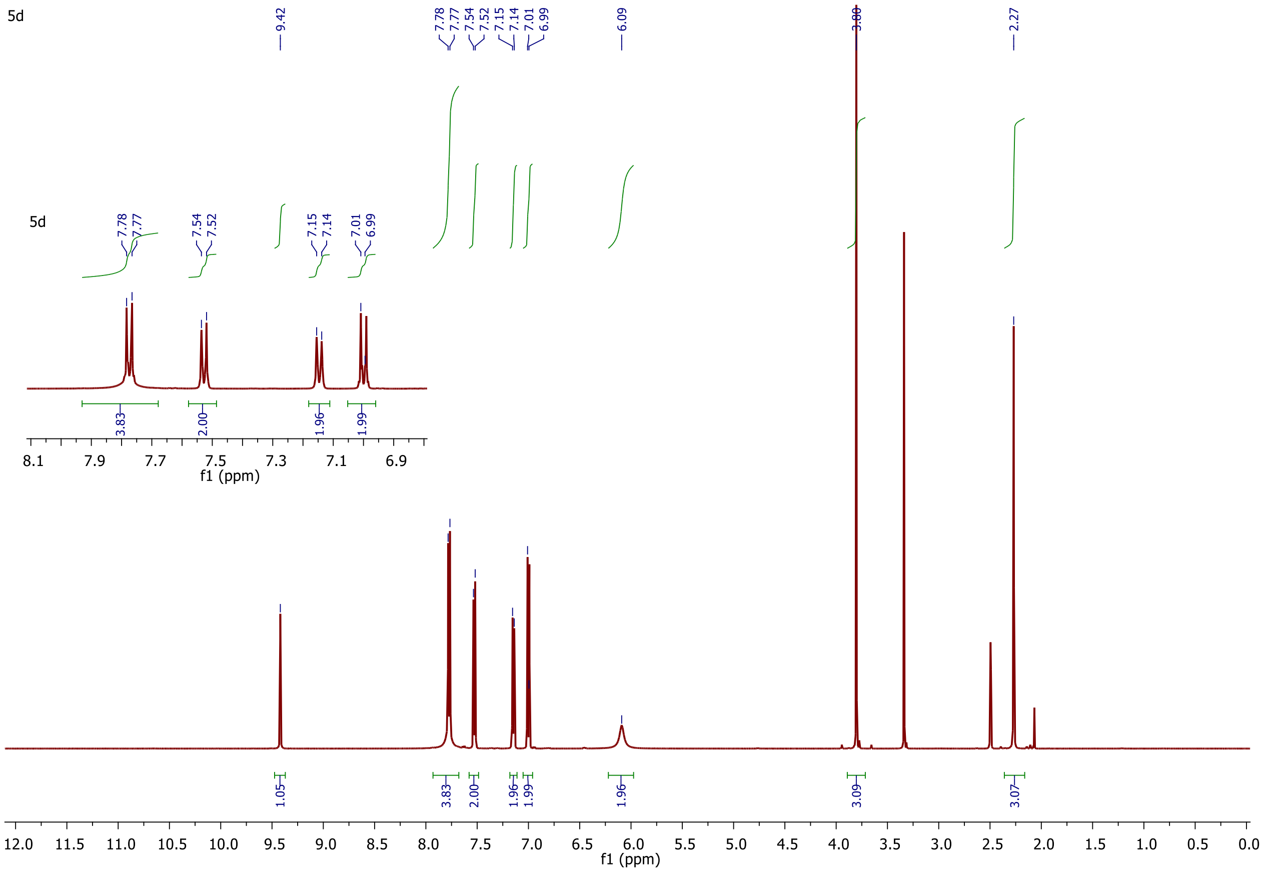

Figure S7: H NMR spectrum of compound **5d** (500 MHz, in DMSO-d_6).

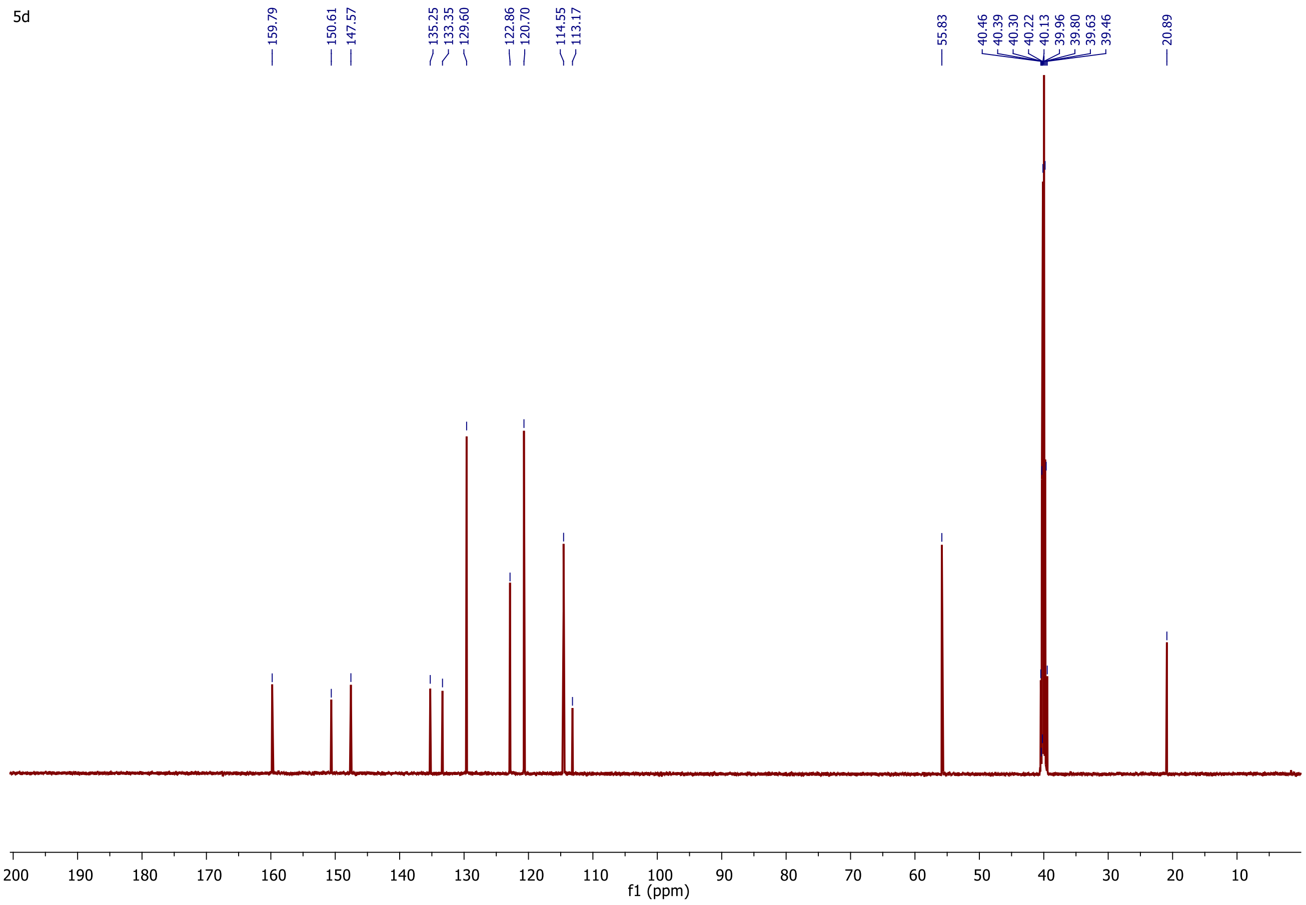

Figure S8: C NMR spectrum of compound **5d** (125 MHz, in DMSO-d_6).

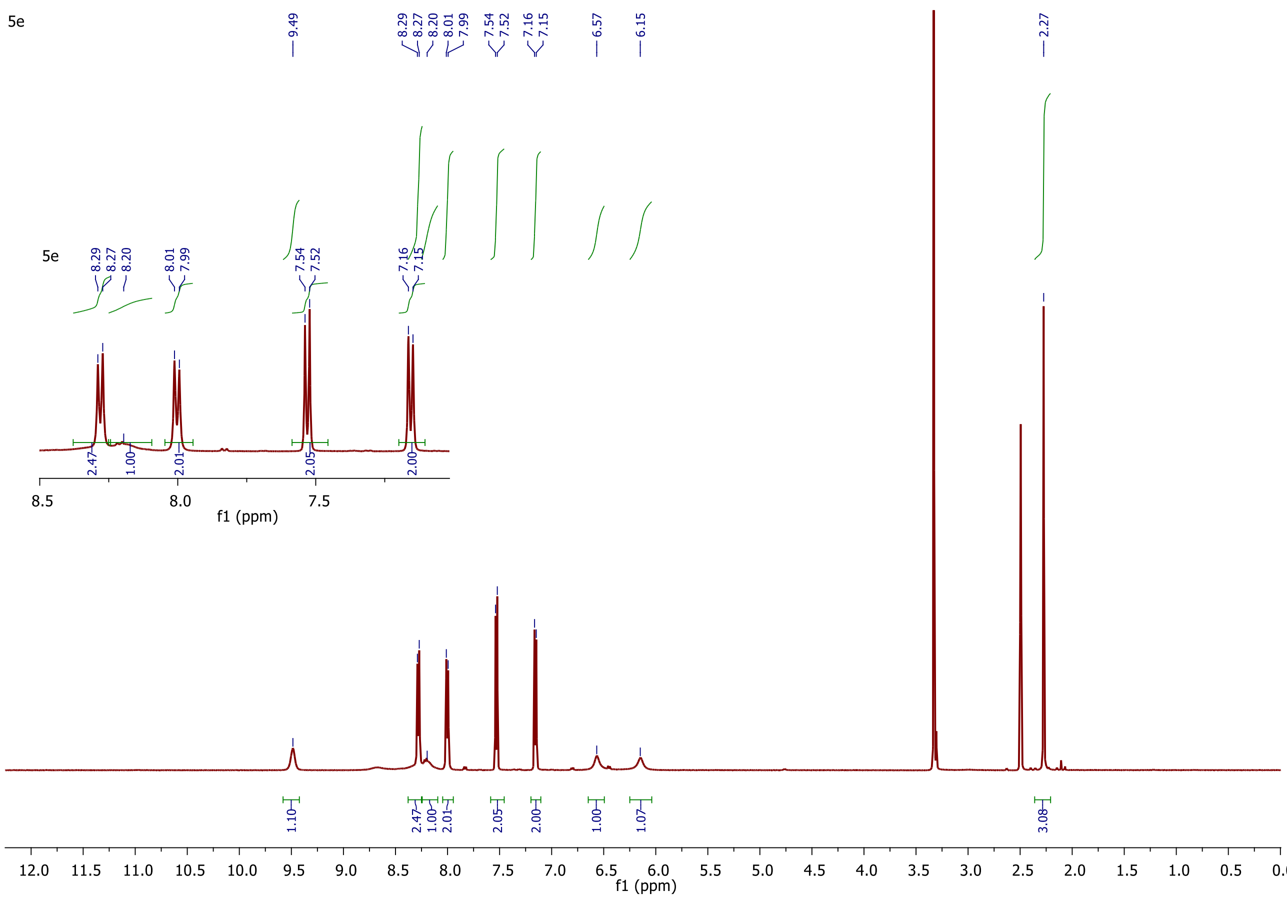

Figure S9: H NMR spectrum of compound **5d** (500 MHz, in DMSO-d_6).

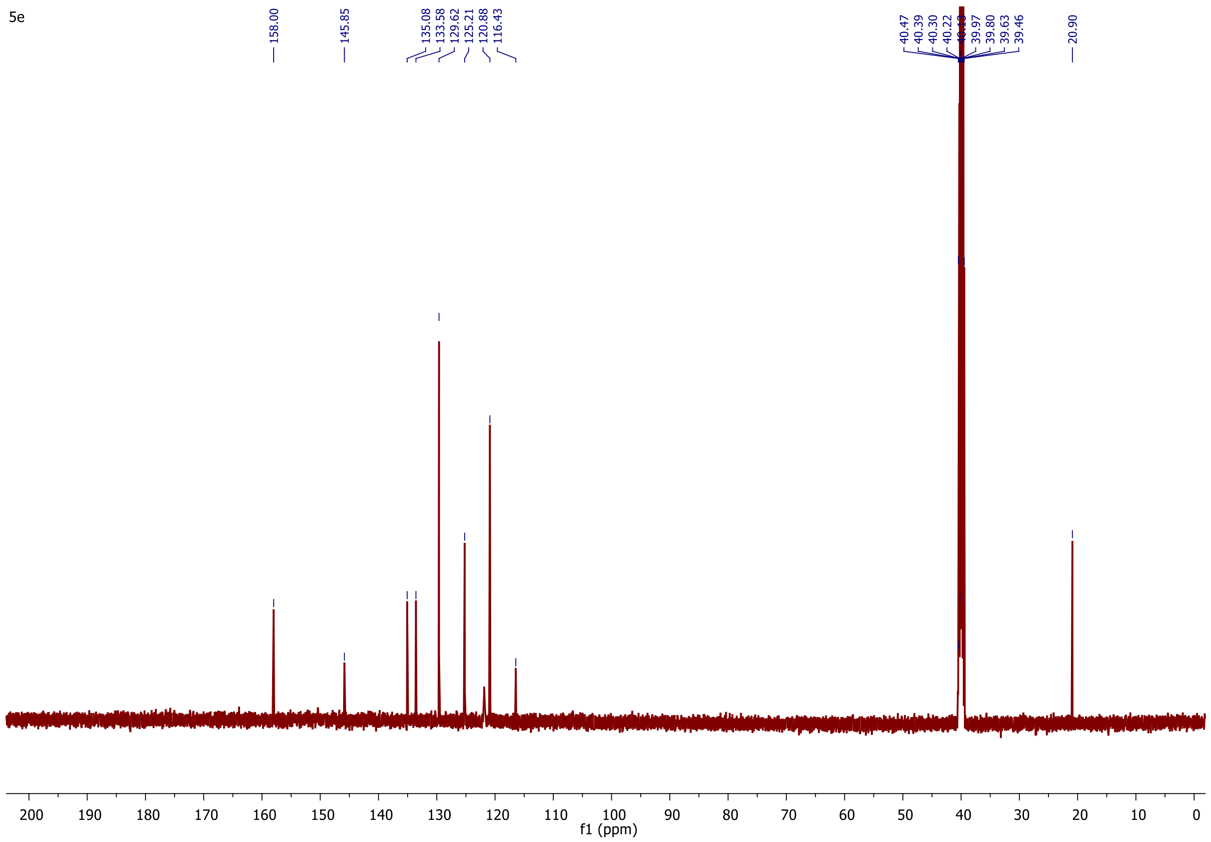

Figure S10: C NMR spectrum of compound **5e** (125 MHz, in DMSO-d_6).

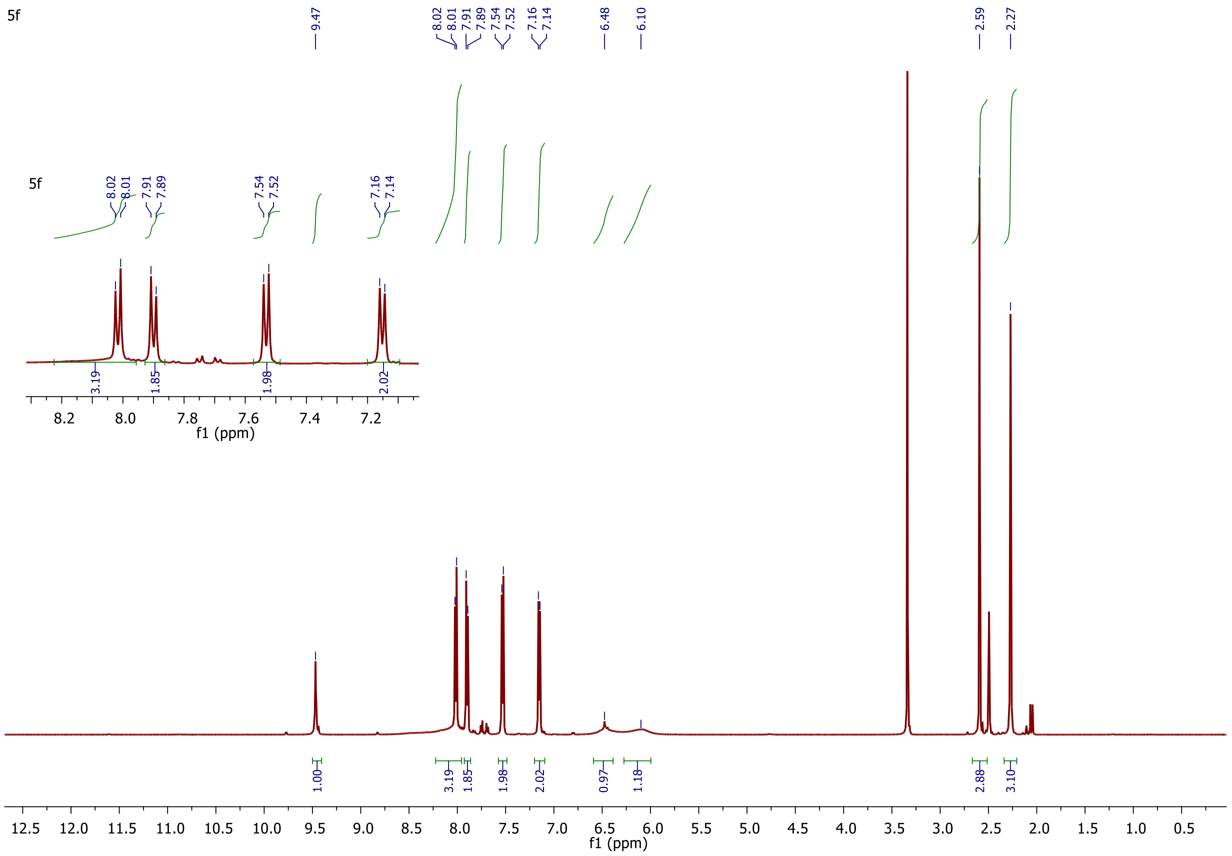

Figure S11: H NMR spectrum of compound **5f** (500 MHz, in DMSO-d_6).

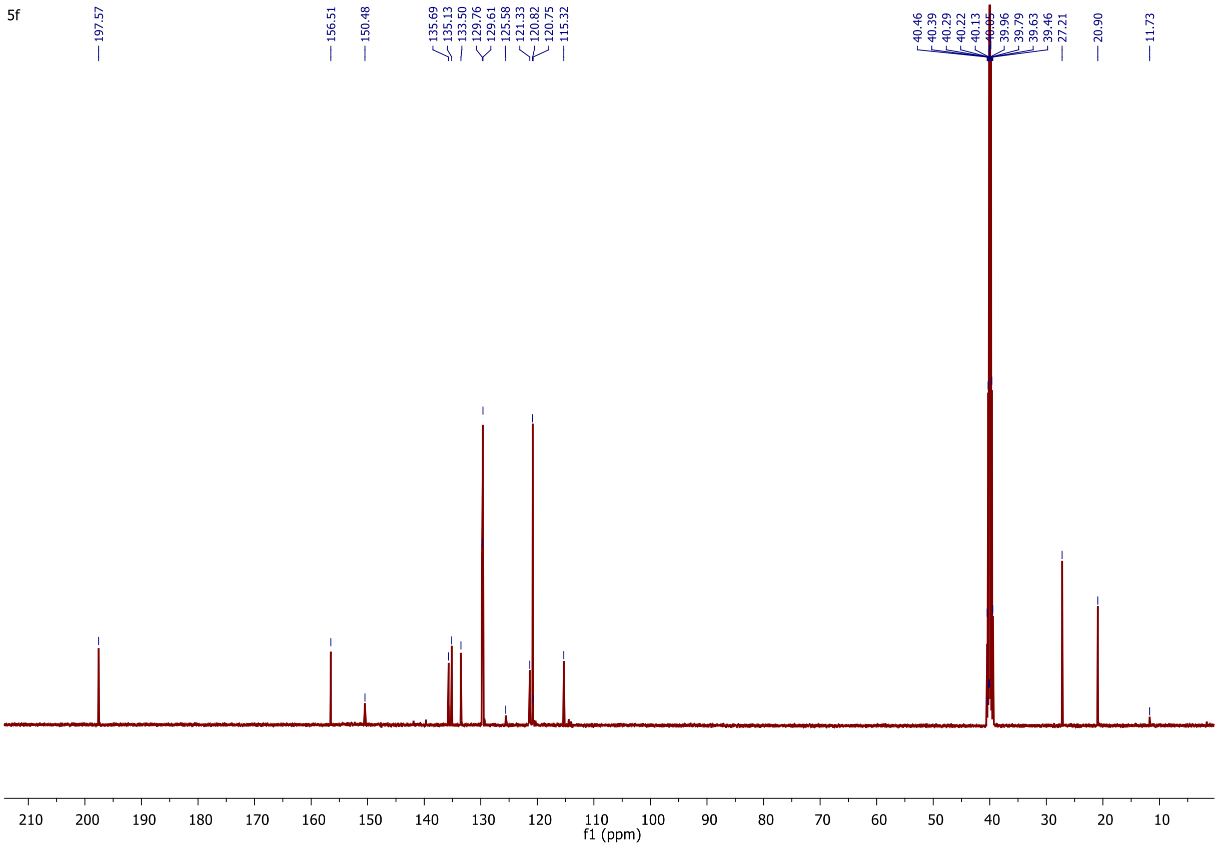

Figure S12: C NMR spectrum of compound **5f** (125 MHz, in DMSO-d_6).

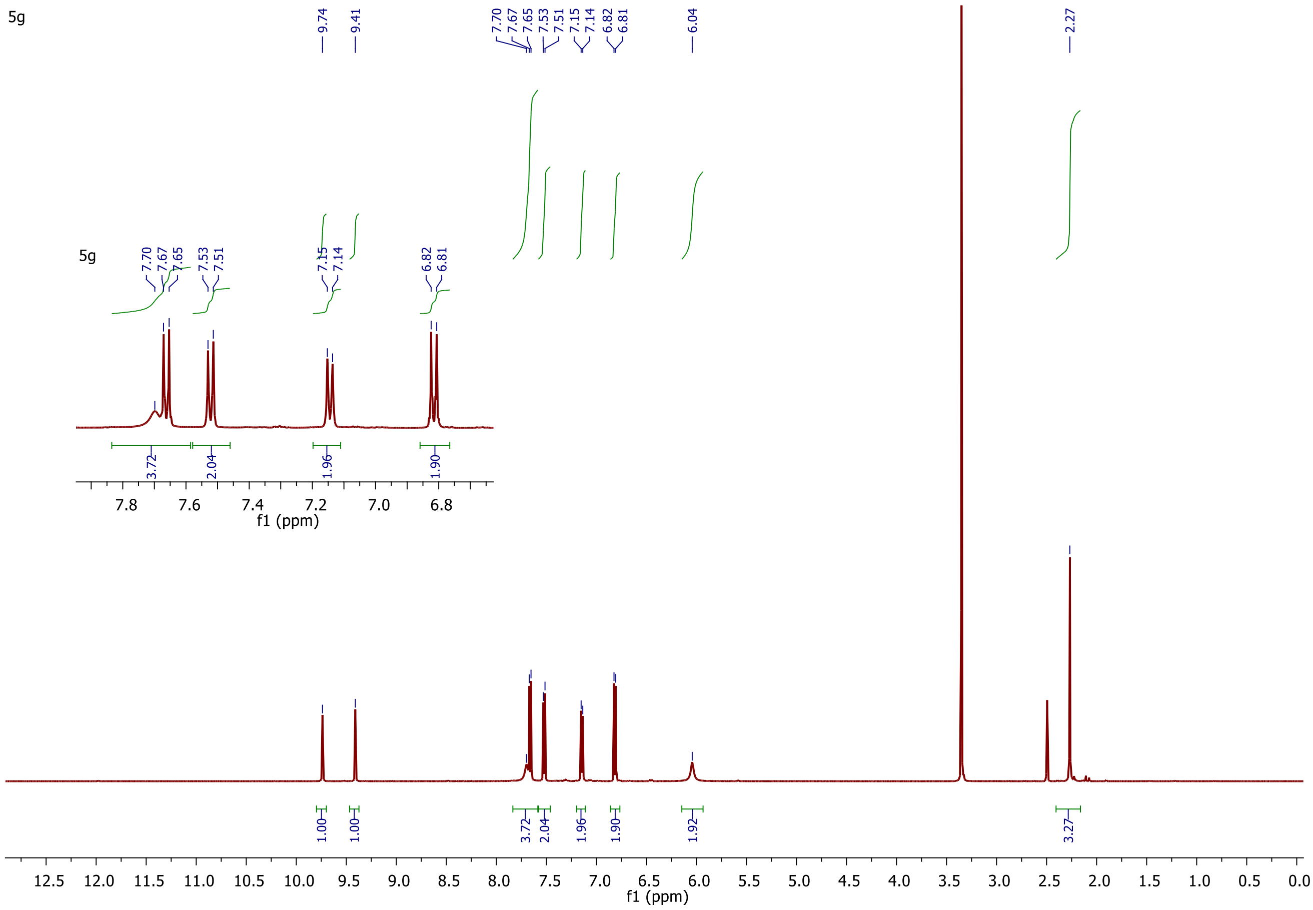

Figure S13: H NMR spectrum of compound **5g** (500 MHz, in DMSO-d_6).

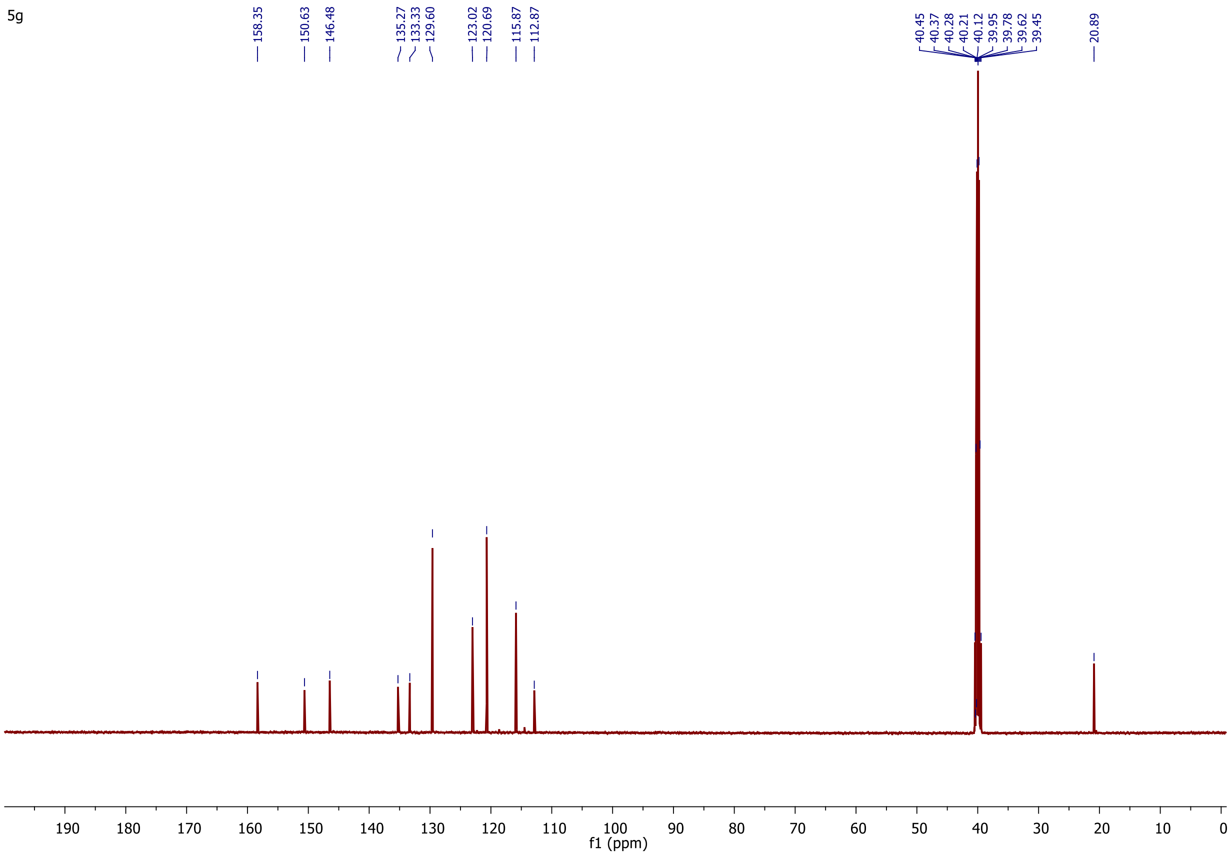

Figure S14: C NMR spectrum of compound **5g** (125 MHz, in DMSO-d_6).

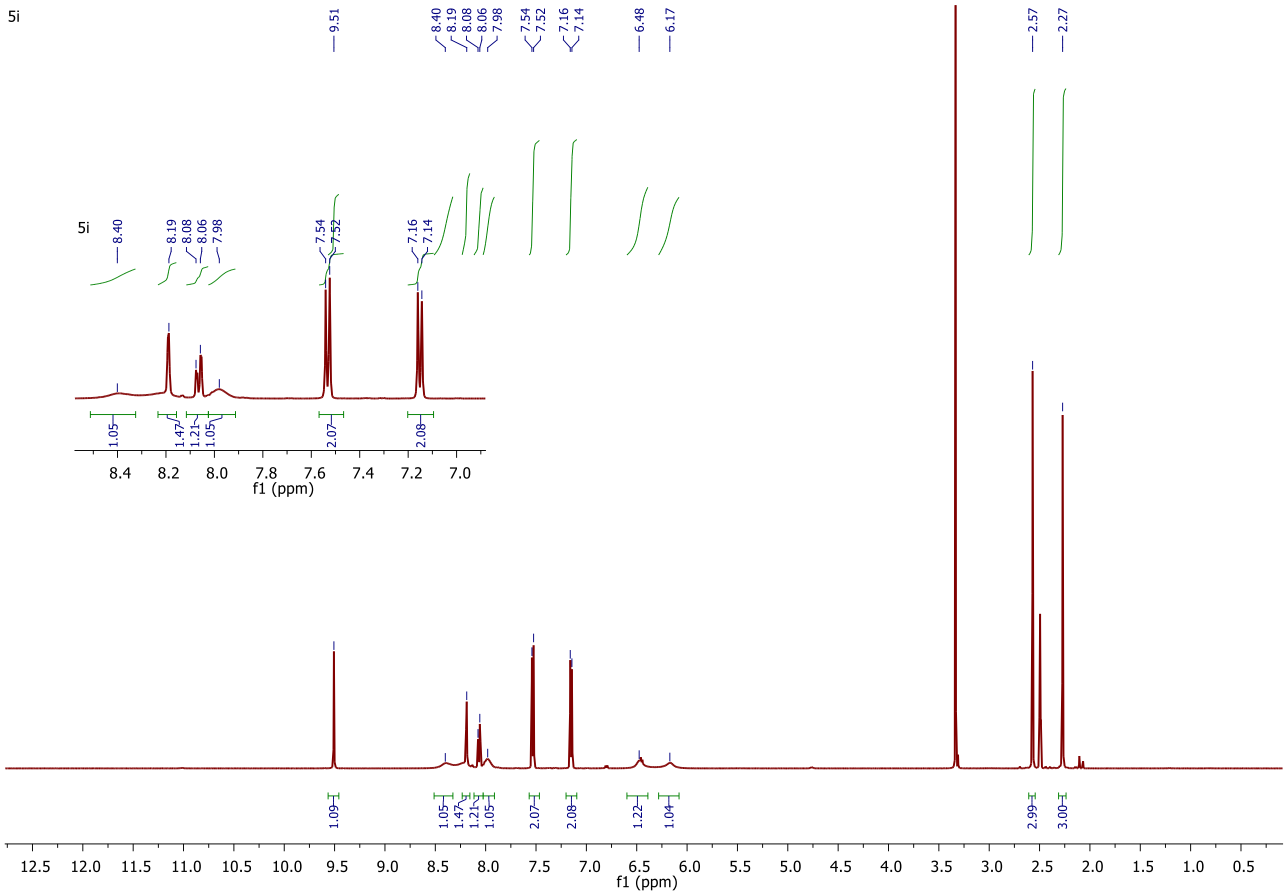

Figure S15: H NMR spectrum of compound **5i** (500 MHz, in DMSO-d_6).

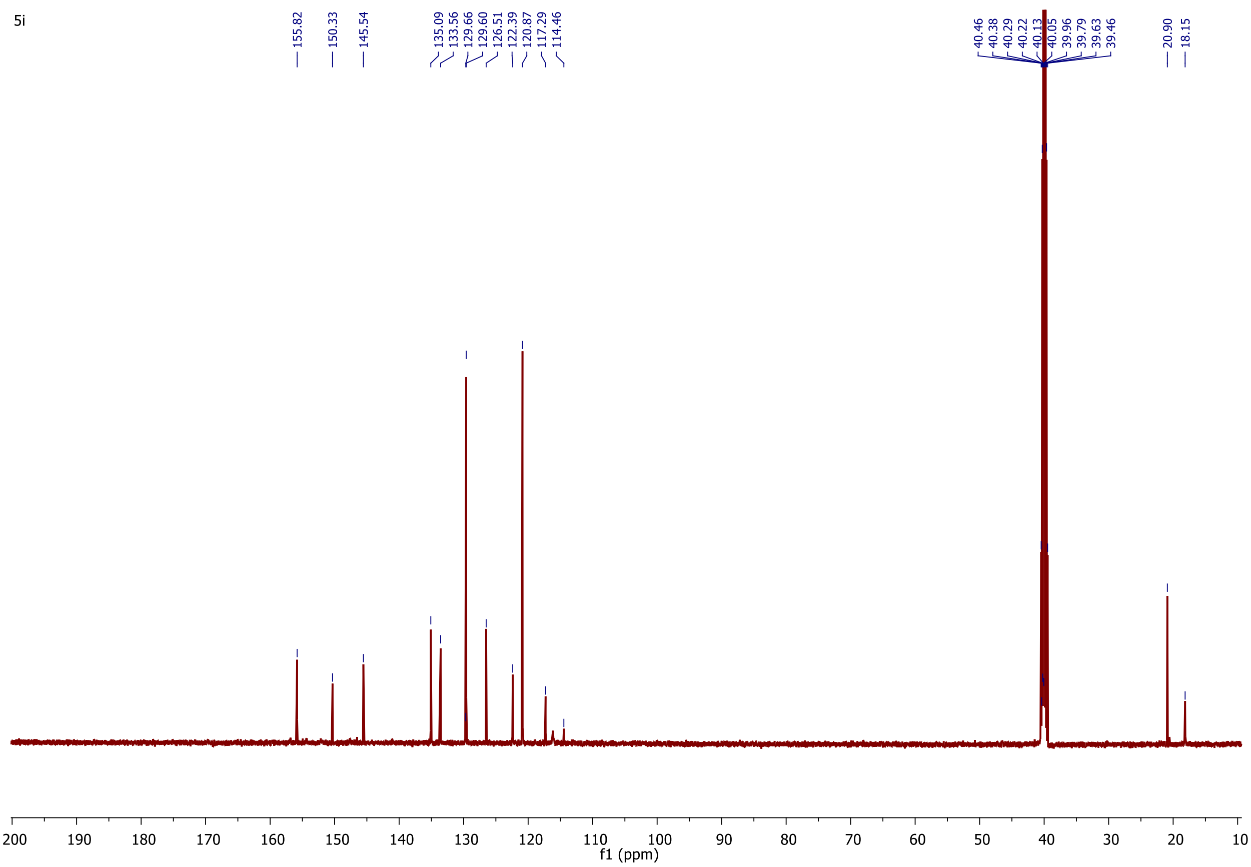

Figure S16: C NMR spectrum of compound **5i** (125 MHz, in DMSO-d_6).

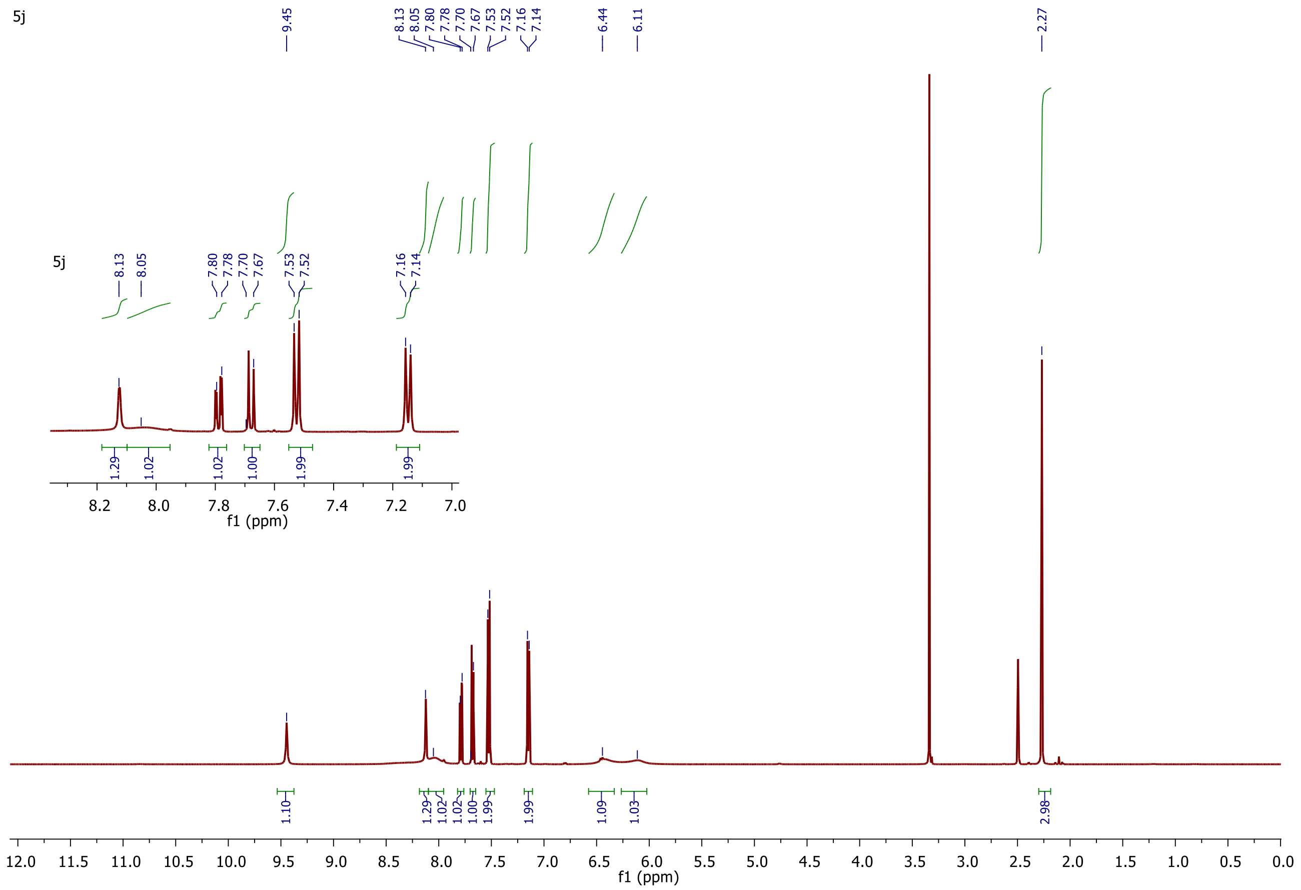

Figure S17: H NMR spectrum of compound **5j** (500 MHz, in DMSO-d_6).

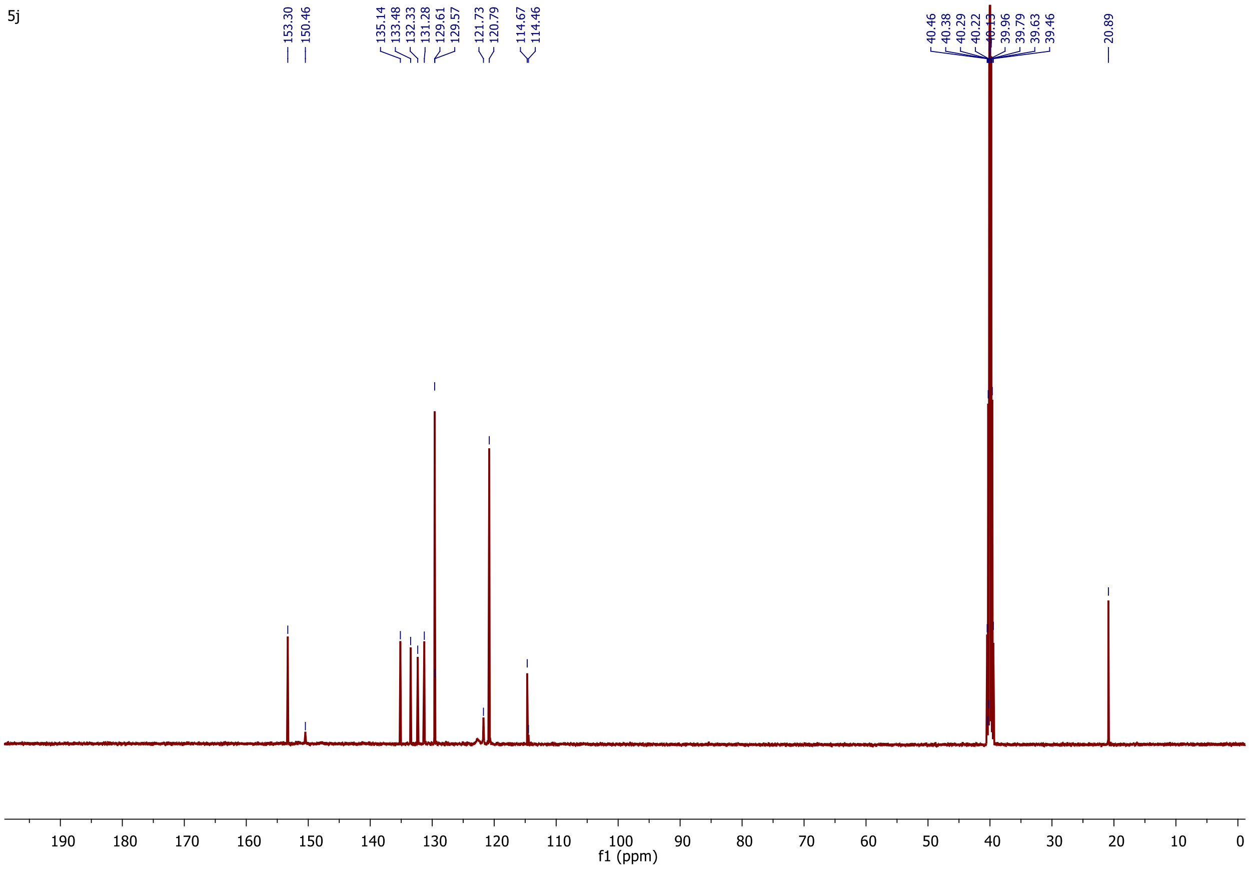

Figure S18: C NMR spectrum of compound **5j** (125 MHz, in DMSO-d$_6$).

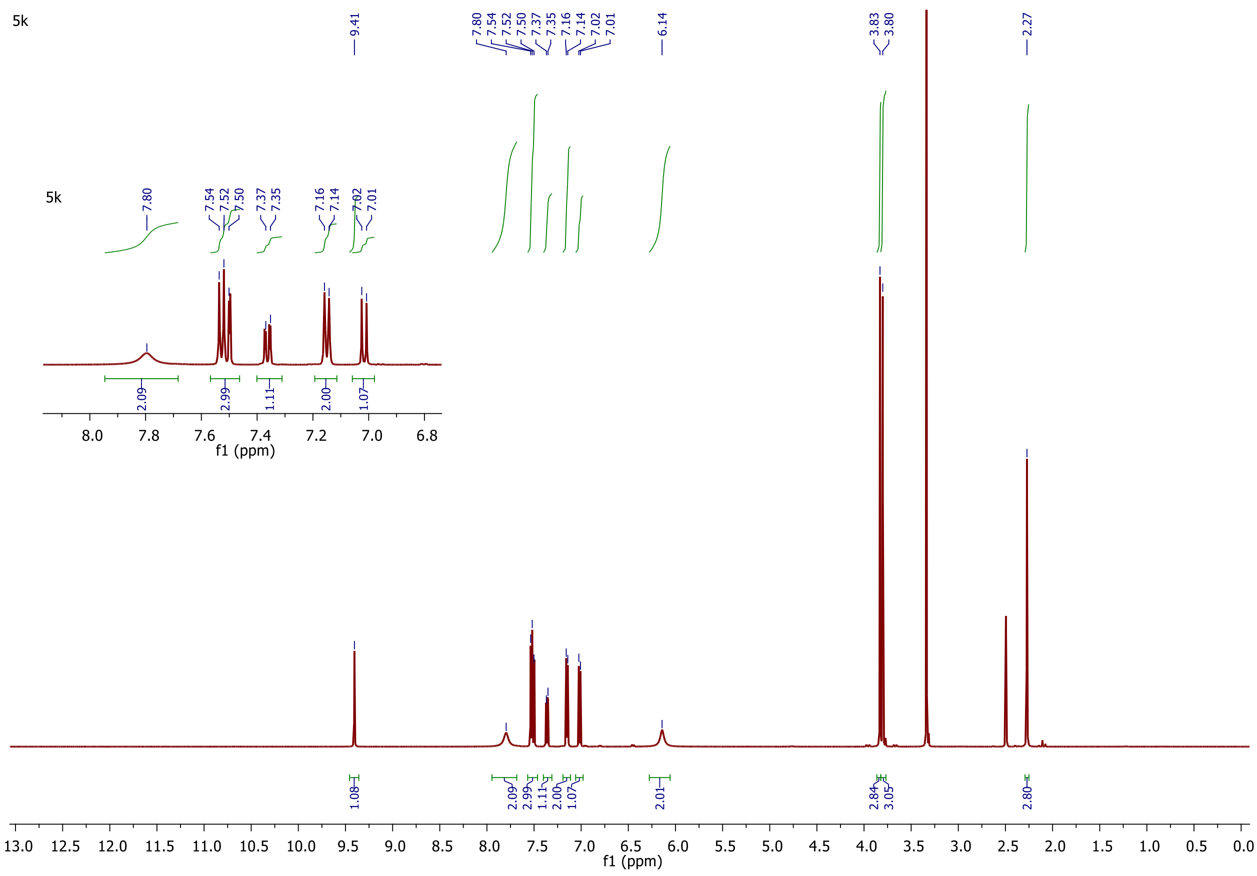

Figure S19: H NMR spectrum of compound **5k** (500 MHz, in DMSO-d$_6$).

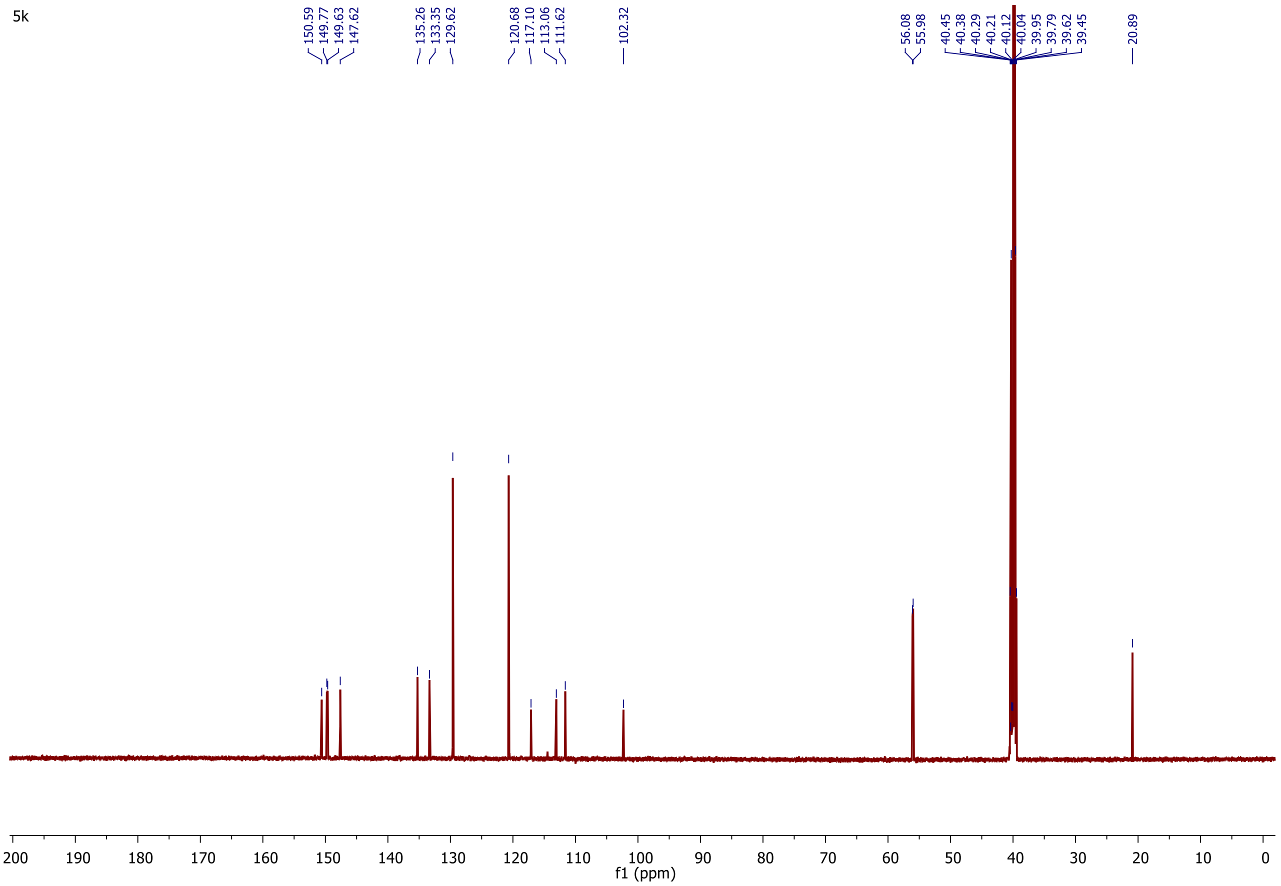

Figure S20: C NMR spectrum of compound **5k** (125 MHz, in DMSO-d_6).

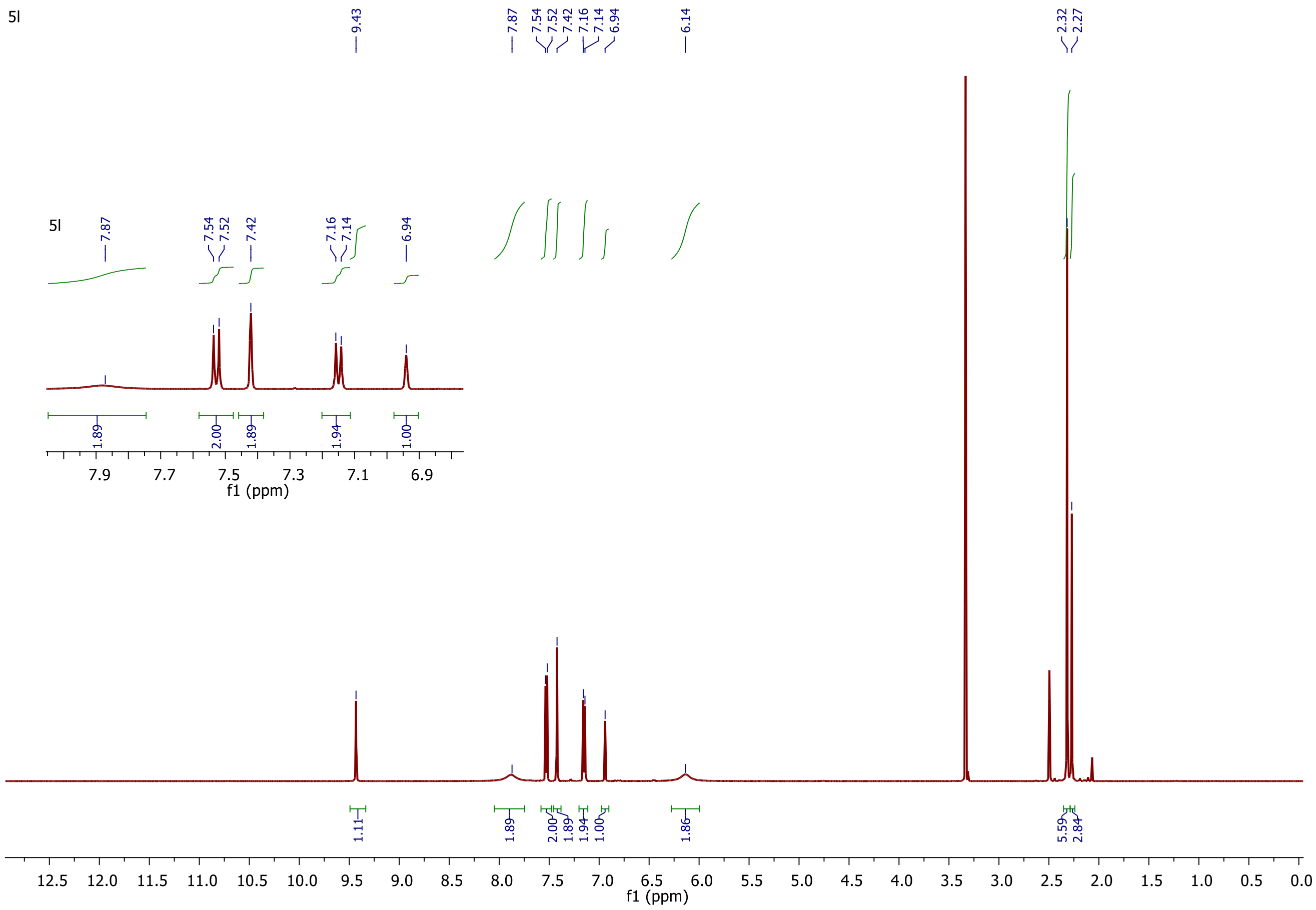

Figure S21: H NMR spectrum of compound **5l** (500 MHz, in DMSO-d$_6$).

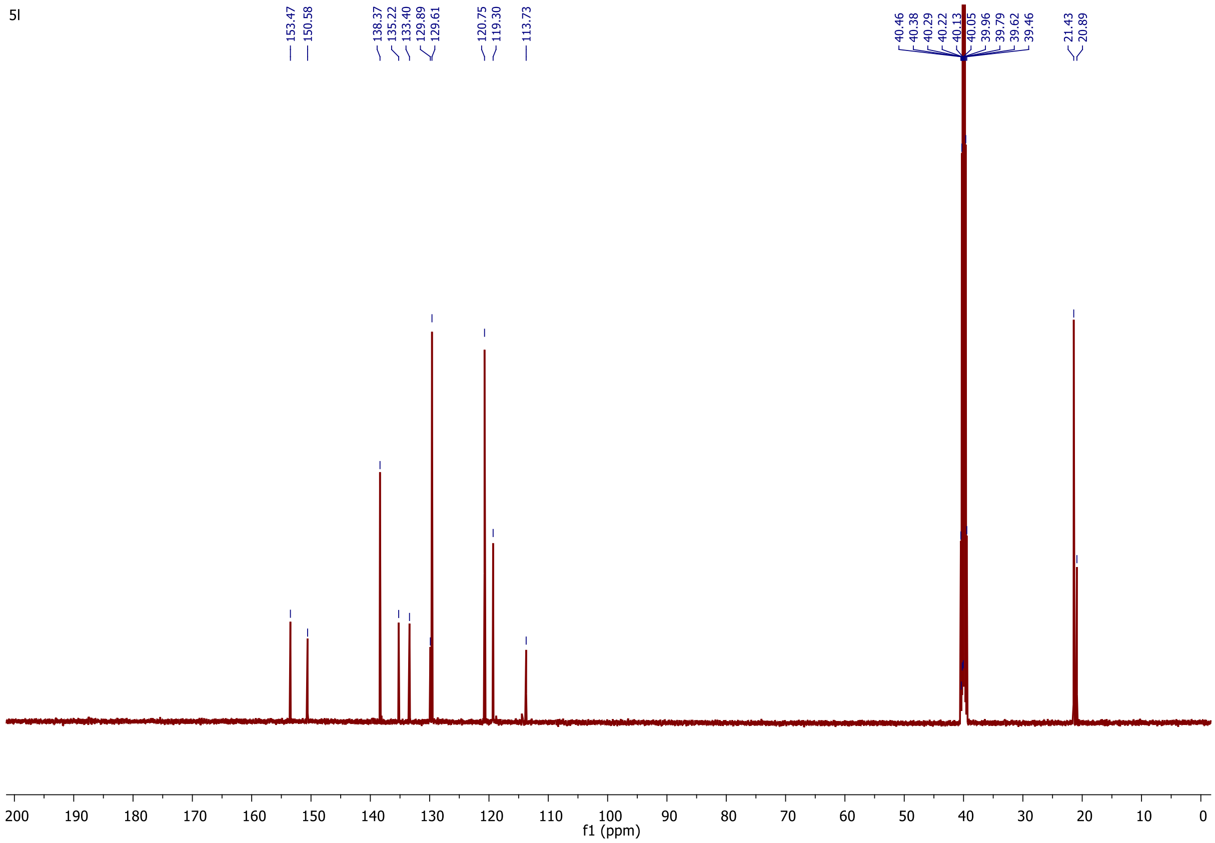

Figure S22: C NMR spectrum of compound **5l** (125 MHz, in DMSO-d_6).
